# VSIG4 as a tumor-associated macrophage marker predicting adverse prognosis in diffuse large B-cell lymphoma

**DOI:** 10.3389/fimmu.2025.1567035

**Published:** 2025-06-05

**Authors:** Dongshen Ma, Yubo Wang, Qingqing Shen, Xinyu Liu, Wenxin Lu, Shaoqi Li, Qianqian Yin, Lei Xia, Guangzhen Liu, Yuhong Chen, Chenxi Xiang, Hui Liu

**Affiliations:** ^1^ Department of Pathology, The Affiliated Hospital of Xuzhou Medical University, Xuzhou, Jiangsu, China; ^2^ Department of Pathology, Xuzhou Medical University, Xuzhou, Jiangsu, China; ^3^ Shanghai Labway Clinical Laboratory Co, Shanghai, China

**Keywords:** diffuse large B-cell lymphoma, tumor microenvironment, tumor-associated macrophages, VSIG4, prognostic markers

## Abstract

**Introduction:**

Diffuse large B-cell lymphoma (DLBCL) exhibits heterogeneous tumor microenvironment. However, the role of tumor-associated macrophages (TAMs) in the DLBCL tumor microenvironment remains unclear. This study aims to elucidate the heterogeneity of TAMs in DLBCL to identify critical TAM-associated prognostic biomarkers.

**Methods:**

Transcriptome data from DLBCL patients were obtained from online database. The CIBERSORT algorithm was applied to quantify TAM abundance across samples. Consensus clustering was used to stratify DLBCL into distinct clusters based on TAM subtype enrichment. Differential gene expression analysis, LASSO regression, univariate/multivariate Cox regression and Kaplan-Meier survival analysis were employed to identify key prognostic biomarkers. Validation of VSIG4+TAM subpopulation was performed using flow cytometry and multiplex immunohistochemistry. A local cohort of 375 DLBCL patients was investigated to explore the correlation between VSIG4 expression and various genetic and pathological characteristics including prognostic outcomes.

**Results:**

Four distinct DLBCL clusters, each enriched with specific TAM subtypes were found. The cluster dominated by M2 TAMs exhibited the worst prognosis. Differential analysis identified VSIG4 as a critical prognostic factor, with high expression in the M2 TAM-enriched cluster. Flow cytometry and mIHC confirmed VSIG4+ TAMs as a subpopulation within CD68+/CD163+ M2 macrophages. VSIG4 expression correlated with adverse genetic features (PIM1, ETV6, CD70 mutations) and aggressive pathological characteristics (non-GCB phenotype, MYC+/BCL-2 double-expression). Multivariate Cox regression confirmed VSIG4 as an independent prognostic factor for poor survival. Survival analysis suggested that VSIG4’s prognostic impact operates independently of regulating lymphocyte infiltration, highlighting its unique role in DLBCL tumor microenvironment.

**Discussion:**

This study identifies VSIG4 as a TAM-associated marker of adverse prognosis of DLBCL and the expression of VSIG4 is related to high-risk genetic and pathological features. These findings position VSIG4 as a promising therapeutic target for immune checkpoint intervention in DLBCL.

## Introduction

Diffuse large B-cell lymphoma (DLBCL) is the most common B-cell malignancy. Although immunochemotherapy based on the combination of rituximab, cyclophosphamide, doxorubicin, and prednisone (R-CHOP) has improved the overall prognosis, 40-50% of the patients could not reach long-term remission and show dismal prognosis due to refractory and relapse diseases (1).

The tumor microenvironment (TME) of DLBCL exhibits significant heterogeneity, mainly resulted from differential cell compositions, cell states and cell-to-cell interactions (2), and has been identified to be essential for the cellular ecosystems, which is closely related to the cell-of-origin (COO), pathogenesis, genetic subtype and prognosis of DLBCL (3, 4). It has been reported that inflammatory cells and stromal cells show significant relationship with immune evasion and eventually effect on drug response and prognosis (5, 6).

Tumor associated macrophages (TAMs), as a vital component of TME, act a pivotal role in tumor immunology and associate with immunotherapy response (7, 8). In the context of TME, TAMs can be polarized to a pro-tumor M2-like phenotype which may enhance immunosuppression and promote tumor growth (9). The significance of TAMs in the microenvironment of DLBCL has not been fully clarified. Although with controversy, it has been reported that CD68+ cells and CD163+ M2-like macrophages may have relevance with tumor progression and poor prognosis (10–14). However, the existing studies are mostly based on relatively small cohorts, and research on the markers for identifying clinically significant subgroups of TAM is still needed.

In this study, we employed bioinformatics methods to identify prognostic markers that are associated with M2-like TAM, leading to the discovery of a macrophage-derived surface marker, VSIG4 (V-set and immunoglobulin domain containing 4), which has the potential to predict unfavorable prognosis of DLBCL. VSIG4, also known as CRIg, is considered as a novel immunoglobulin superfamily member and acts as a complement receptor (15). VSIG4 can potentially regulate macrophage functions and shows immunosuppression effects (16, 17). In the microenvironment of some solid tumor, VSIG4+ TAMs are related to poor prognosis potentially by impairing anti-tumor T cell activity (18–20), and may serve as an immune checkpoint target (21). Previously, it has been reported that VSIG4 is highly expressed in T cell/histiocyte-rich large B-cell lymphoma (22) and Epstein-Barr virus positive post-transplant DLBCL (23), but still lacking the systematic study of VSIG4 in DLBCL. Here, we aim to clarify the expression pattern and clinical significance of VSIG4 to demonstrate its value as an immune checkpoint target in DLBCL.

## Materials and methods

### Patients

In this study, the clinical and pathological information of a total of 375 DLBCL patients newly diagnosed in The Affiliated Hospital of Xuzhou Medical University between 2015 and 2021 were retrieved from hospital records. All cases were diagnosed according to the WHO criteria and reviewed by 2 pathologists specialized in hematological tumors. The exclusion criteria included: (1) DLBCL positive for CyclinD1 expression or *CCND1* translocation, (2) transformed large B-cell lymphoma, (3) infected by human immunodeficiency virus, (4) with multiple malignant disease. This study was approved by the Ethics Committee of The Affiliated Hospital of Xuzhou Medical University (grant XYFY2024-KL123-01).

### Immunohistochemistry

The immunohistochemistry (IHC) was performed on tissue microarray (TMA) sections according to routine procedure. Each TMA block contained 24–43 tissue cores of tumor area in 2mm^2^ diameter. The primary antibodies used in this work included: VSIG4 (Clone: EPR22576-70, Cat.AB252933), CD68 (Clone: PG-M1, Cat.ZM-0464), CD163(Clone: 10D6, Cat.ZM-0428), CD206(Clone: 2A6A10, Cat.60143-1), MYC (Clone: EP121, Cat.ZA-0555), BCL-2 (Clone: OTIR1H2, Cat.ZA-0536), BCL-6 (Clone: LN22, Cat.ZM-0011), CD10 (Clone: UMAB235, Cat.ZM-0283), MUM-1 (Clone: OTI6F6, Cat.ZM-0401). Except for VSIG4 (Abcam, USA) and CD206 (Proteintech, USA), all antibodies were purchased from ZSGB-BIO (China). The detection system was Polymer HRP-Goat anti-Rabbit/Mouse kit (Cat.PV-8000, ZSGB-BIO, China). VSIG4 and CD206 were considered positive when there were at least 1% of cells observed with convincing DAB signals. The CD68-positive cells and CD163-positive cells were counted and averaged under 3 high-power fields, and cases were divided into CD68 high/low group and CD163 high/low group based on the median number of positive cells. The cutoff values for MYC, BCL-2, BCL-6, CD10, MUM1 were 40%, 50%, 30%, 30% and 30%, respectively (24, 25). All IHC sections were reviewed by 2 pathologists. All images were captured by a BX53 microscope (Olympus, Japan).

Detailed information can be found in **Supplementary Methods** for bioinformatic study, single-cell transcriptome analysis, fluorescence *in-situ* hybridization (FISH), multiplex IHC, flowcytometry, COO analysis by Lymph2Cx, next generation sequencing (NGS), LymphGen subtyping and statistical analysis.

## Results

### DLBCL enriched with M2 TAMs showed unfavorable prognosis

**Figure 1:**
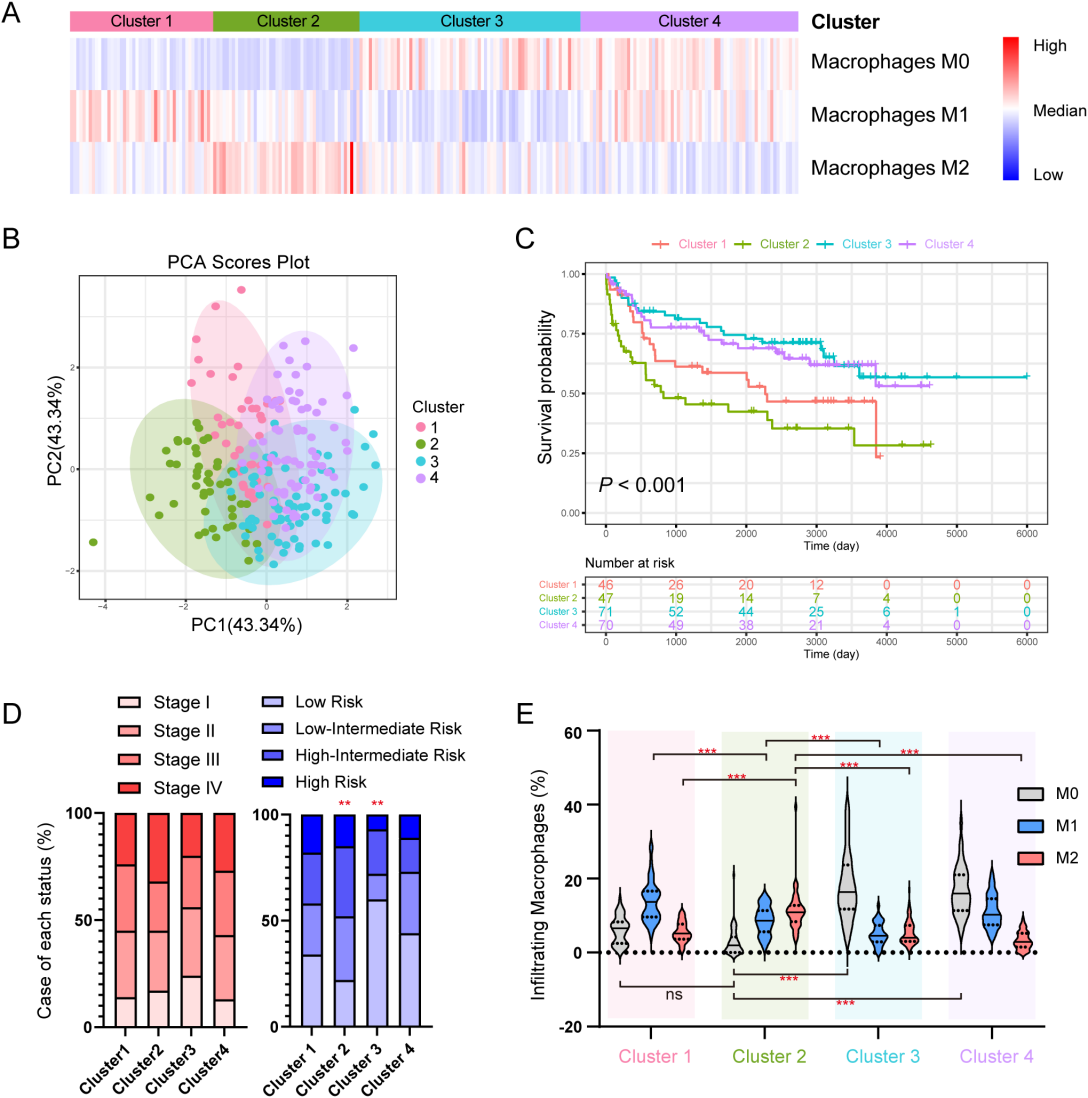
Subclusters of DLBCL classified by the abundance of TAMs derived from CIBERSORT. **(A)** Heatmap of the consensus clustering of the DLBCL cases by the infiltrating abundance of M0, M1 and M2 macrophages. **(B)** PCA analysis of the clusters. **(C)** K-M plot and survival analysis of 4 clusters. **(D)** Case distribution of clinical stage (left) and IPI risk (right) in each cluster. **(E)** Abundance of infiltrating macrophages (%) in each cluster. * indicates *P*-value <0.05; ** indicates *P*-value <0.01; *** indicates *P*-value <0.001; ns indicates “not significant”.

To confirm whether DLBCL cases can be further classified by different types of TAMs, we applied CIBERSORT assay to a 234-case online dataset to quantify the infiltrating abundance (%) of immune cells and used the relative abundance of TAMs (M0, M1 and M2) for consensus clustering. Four clusters were identified (**Figure 1A**): Cluster 1 with elevated M1 TAMs, Cluster 2 with increased M2 TAMs, Cluster 3 dominated by undifferentiated M0 TAMs, and Cluster 4 with a higher abundance of both M0 and M1 TAMs. Principal component analysis (PCA) analysis revealed distinct infiltration patterns of immune cells (**Figure 1B**), and the overall survivals (OS) varied among the clusters, with Cluster 2 (M2-enriched) showing the poorest (**Figure 1C**).

No significant difference in the distribution of cases was observed across clinical stages and clusters (**Figure 1D** **, left**), however, patients with low IPI risk were less frequent in Cluster 2 but more distributed in Cluster 3, (**Figure 1D** **, right**). Cluster 2 had a higher M2 level compared to other clusters, and a lower M1 level compared to Cluster 3 and Cluster 4 (**Figure 1E**). **Supplementary Figure S1** compared the abundance of other immune cell types across the four clusters.

We observed no abundance differences of M0 or M1 but a significant difference of M2 between female and male patients (**Supplementary Figure S2A**). M0 TAMs negatively correlated with age, while M2 TAMs positively correlated (**Supplementary Figure S2B**). M0 or M2 TAMs did not differ for clinical stages but M1 TAMs were significantly less abundant in stage I patients (**Supplementary Figure S2C**). No significant associations were found between TAMs and IPI risks (**Supplementary Figure S2D**).

### VSIG4 is a potential marker for poor prognosis differentially expressed in M2-enriched cluster

**Figure 2:**
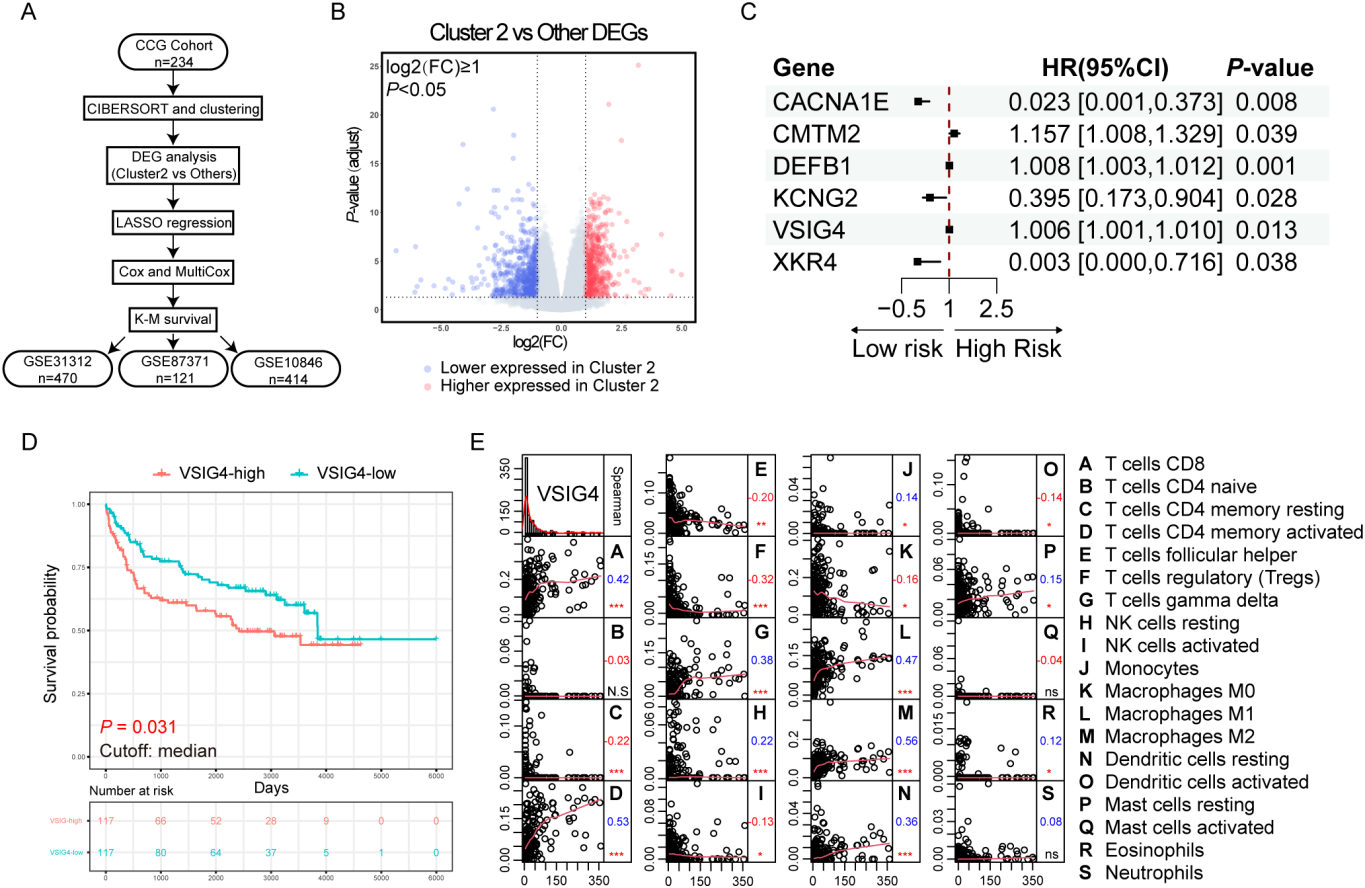
Discovering the unfavorable prognostic marker upregulating in the cluster enriched with M2-like TAMs. **(A)** Flowchart of the analysis process. **(B)** Volcano plot of the differentially expressed genes in cluster 2. **(C)** Forest plot of the results of multivariate Cox regression. **(D)** K-M plot and survival analysis of VSIG4-High group versus VSIG4-Low group. **(E)** Correlations between the expression of VSIG4 and the infiltration of tumor microenvironmental cells. Numbers below the labels indicate the correlation coefficients; * indicates *P*-value <0.05; ** indicates *P*-value <0.01; *** indicates *P*-value <0.001; N.S indicates “not significant”.

To pinpoint the crucial prognostic genes linked to Cluster 2, we employed a comprehensive set of bioinformatics analysis and subsequently validated our findings in three independent GEO cohorts (**Figure 2A**). A total of 1063 genes were identified as differentially expressed between Cluster 2 and other clusters (**Figure 2B**). Gene oncology (GO) analysis and Kyoto Encyclopedia of Genes and Genomes (KEGG) analysis of these differentially expressed genes (DEGs) were shown in (**Supplementary Figures S3A, B**). Gene Set Enrichment Analysis (GSEA) analysis revealed that Cluster 2 exhibited a higher enrichment of genes that were up-regulated during M0-M2 polarization (**Supplementary Figure S3C**).

We performed LASSO regression (λ.min = 0.082, **Supplementary Figure S3D**) and univariate/multivariate Cox regressions to resolve multicollinearity and identify the independent prognostic genes among the DEGs. Six genes were identified with CMTM2, DEFB1 and VSIG4 predicting unfavorable prognosis (HR > 1, *P* < 0.05, **Figure 2C**). Kaplan-Meier (K-M) survival analysis showed only VSIG4 mRNA significantly stratified the OS (*P* = 0.031, **Figure 2D**), and its prognostic impact was confirmed in three array-based mRNA expression datasets (**Supplementary Figures S3E–G**), linking high expression of VSIG4 to worse OS.

VSIG4, a surface marker specifically expressed in macrophages and certain tumor cells, showed positive correlations with M1 and M2 macrophages, CD8+ T cells and activated CD4+ memory T cells, γδT cells, but negative correlations with resting CD4+ memory T cells, follicular T helper cells, regulatory T cells, monocytes and M0 macrophages in the TME (**Figure 2E**). It was also enriched in the biological progress of “humoral immune response” (**Supplementary Figure S3H**), suggesting a role for VSIG4 in tumor immunology. The differences in clinical pathological characteristics between VSIG4-high and VSIG4-low cases in NCICCR, GSE31312, GSE87371, GSE10846 cohorts were respectively summarized in **Supplementary Tables S1**-**S4**.

### VSIG4 is mainly expressed on a subset of CD68+/CD163+ TAMs of DLBCL

**Figure 3:**
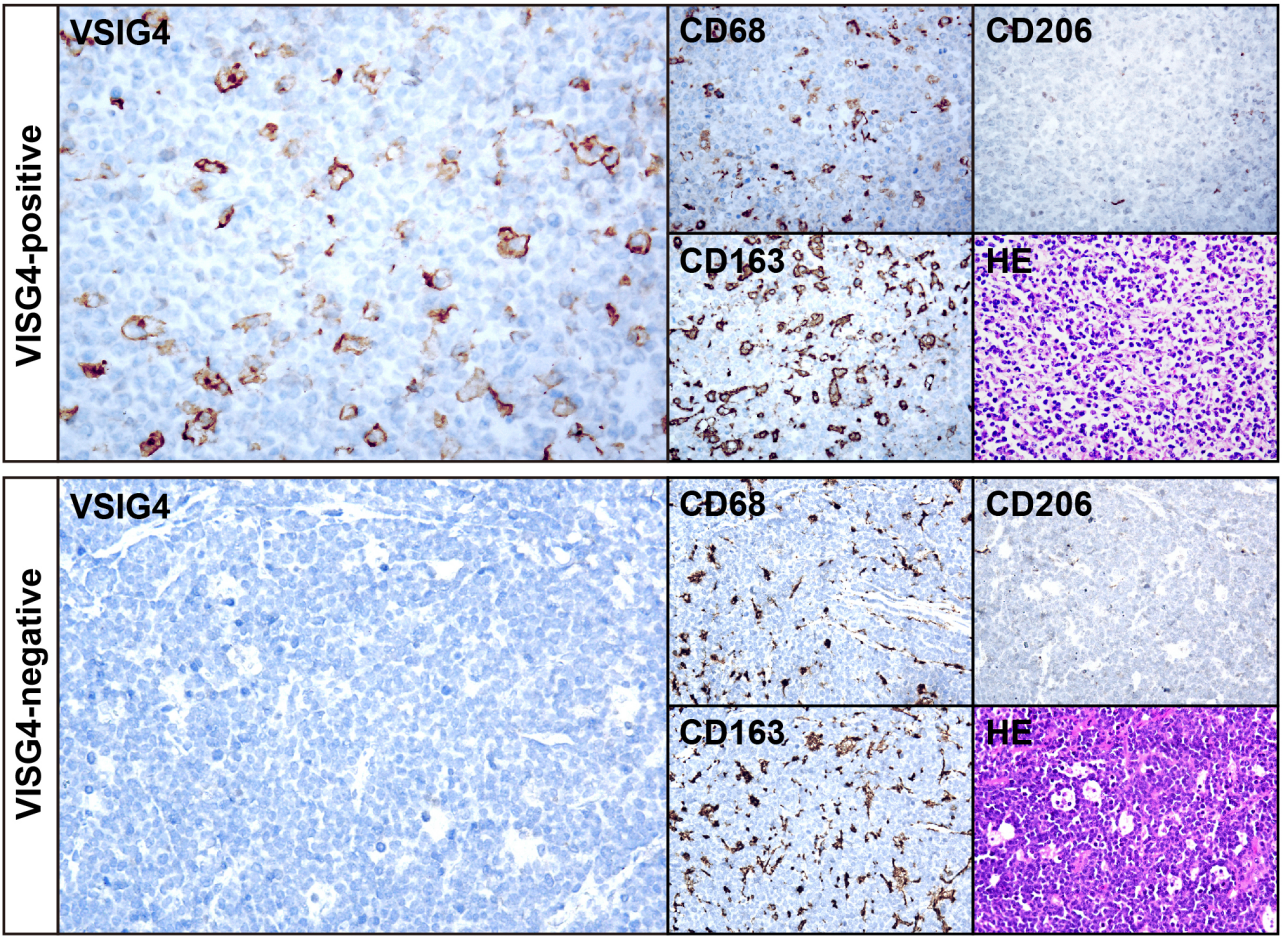
IHC results of representative cases of VSIG4+ and VSIG4- DLBCL. The images of VSIG4, CD68, CD163, CD206 and HE of a representative VSIG4+ case (top) and a representative VSIG4- case (bottom). Original magnification ×400.

We utilized IHC on TMA sections containing 375 cases to assess VSIG4 expression and classical macrophage markers CD68, CD163 and CD206. Representative examples of a VSIG4+ case and a VSIG4- case were shown in **Figure 3**. Like CD68, CD163 and CD206, VSIG4 exhibited focal or diffuse membrane staining on the macrophages. In our cohort, 63.7% and 76.0% of the cases were VSIG4 and CD206 positive, respectively, while CD63+ or CD168+ cells could be observed in nearly all cases.

**Figure 4:**
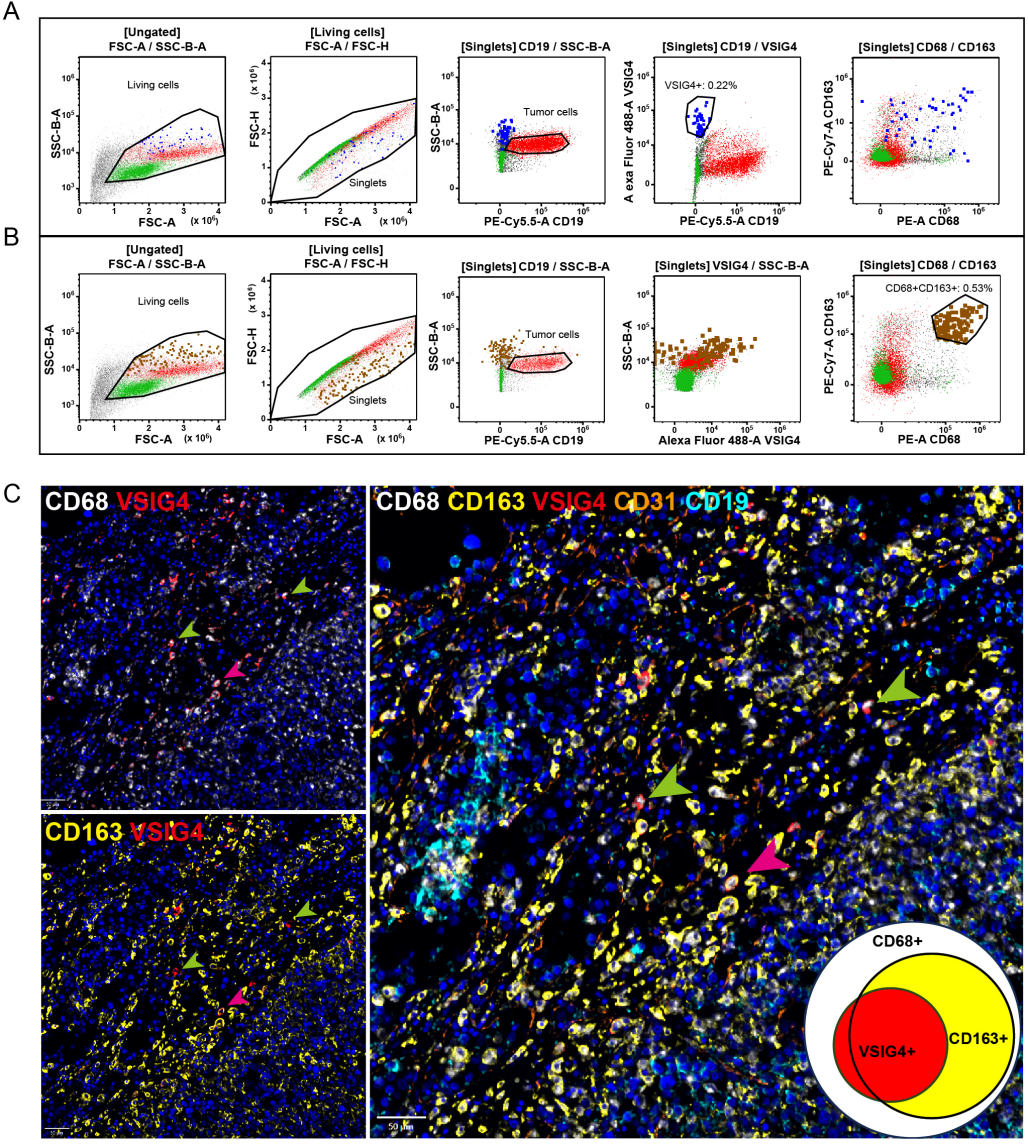
The co-expression of VSIG4 with TAM markers. **(A)** Flowcytometry analysis of a DLBCL cases showing the VSIG4 was expressed mainly in CD68+/CD163+ cells and **(B)** a part of but not all CD68+/CD163+ cells were VSIG4+. VSIG4+ cells were marked blue in **(A)** and CD68+/CD163+ cells were marked brown in **(B)**. **(C)** Multiple IHC images of a representative VISG4+ case showing most majority of VSIG4+ cells co-expressed with CD68+/CD163+ cells (pink arrows) but still some were CD68+/CD163- (green arrows). Original magnification ×400 for panel C.

To gain a comprehensive understanding of the expression pattern of VSIG4 within the TME of DLBCL, flowcytometry was performed on a patient’s neoplastic lymph node. We found VSIG4+ cells constituted 0.22% of all living singlets and were largely CD68+ or CD68/CD163 double positive (**Figure 4A**). By gating typical CD68+/CD163+ M2-like cells we observed a smeared VSIG4 expression pattern in this group (**Figure 4B**).

Furthermore, we performed multiplex IHC on three representative cases to verify the co-expressions of VSIG4 with other macrophage markers. **Supplementary Figure S4A** showed a case with diffuse VSIG4 expression, closely matching CD68+/CD163+ cell distribution. In this case, most CD68+/CD163+ cells co-expressed VSIG4. However, we did observe CD68+/VSIG4+/CD163- cells in a case with VSIG4 focally distributed (**Figure 4C**, green arrow), although most VSIG4+ cells were triple-positive (**Figure 4C**, pink arrow). Notably, VSIG4 was expressed only on a subset of CD68+/CD163+ cells in this case. **Supplementary Figure S4B** showed a case lacking VSIG4+ cells, with only CD68+ and CD163+ cells present. We noticed that in all three cases, nearly all macrophages displayed CD68+/CD163+ M2-like phenotype and no VSIG4 co-expression with CD19+ tumor cells or CD31+ endothelial cells. These findings suggested VSIG4 as a marker for a specific subset of TAMs, which accounted for a variable proportion of CD68+/CD163+ cells and a small proportion of CD68+/CD163- cells.

### Clinical and pathological characterization of VSIG4+ DLBCL

The clinical and pathological features of VSIG4- cases (n = 136) and VSIG4+ cases (n = 239) were compared (**Table 1**). Clinically, age was the only variable with a significant disparity, showing a higher prevalence of older patients in the VSIG4+ group (59.8% versus 46.3%). No significant differences were found in sex, involvement of extranodal sites, LDH level, clinical stage, IPI score, B symptoms or hypoalbuminemia. Pathologically, VSIG4+ DLBCL had a higher incidence of non-GCB (55.1% versus 37.0%), BCL-2 positivity (68.0% versus 49.5%), MYC+/BCL-2+ double expression (DE, 39.8% versus 23.2%) and MUM1 positivity (68.0% versus 50.7%), but lower incidence of CD10 positivity (26.8% versus 40.7%) and *MYC* translocation (4.3% versus 15%). No differences were observed in MYC expression, BCL-6 expression, KI-67 expression, *BCL2* translocation, *MYC/BCL2* double-hit or *MYC/BCL2/BCL6* triple-hit between the groups.

**Table 1:** Clinicopathologic characteristics of VSIG4- and VSIG4+ patients.

Characteristics	n (%)		*P*
	VSIG4- DLBCL	VSIG4+ DLBCL	
Clinical features			
Age > 60 y	63 (46.3)	143 (59.8)	0.011
Sex: male	73 (53.7)	120 (50.2)	0.518
Extranodal sites (≥2)	14 (10.3)	39 (16.3)	0.107
Elevated LDH	47 (38.8)	102 (45.5)	0.231
Stage (III-IV)	40 (29.6)	89 (38.4)	0.091
IPI (3-5)	25 (20.8)	51 (22.7)	0.915
B symptoms	24 (17.6)	37 (15.7)	0.621
Hypoalbuminemia	54 (40.6)	107 (45.3)	0.378
Pathological features			
Non-GCB	50 (37.0)	130 (55.1)	<0.001
MYC+	39 (52.0)	105 (62.1)	0.159
BCL-2+	53 (49.5)	143 (68.0)	0.001
BCL-6+	121 (91.0)	208 (88.5)	0.448
MYC+/BCL-2+ DE	22 (23.2)	72 (39.8)	0.006
CD10+	55 (40.7)	63 (26.8)	0.006
MUM1+	58 (50.7)	160 (68.0)	<0.001
KI-67 (>90%)	7 (5.3)	9 (4.0)	0.602
*MYC* Translocation	9 (15.0)	4 (4.3)	0.035
*BCL2* Translocation	10 (17.2)	8 (8.5)	0.125
*BCL6* Translocation	11 (19.6)	17 (18.8)	1.000
*MYC/BCL2* DH	4 (6.7)	1 (1.1)	0.078
*MYC/BCL2/BCL6* TH	2 (3.3)	1 (1.1)	0.561
High CD68+ cells	41 (30.1)	145 (60.7)	<0.001
High CD163+ cells	28 (20.6)	159 (66.5)	<0.001
CD206+	93 (68.4)	192 (80.3)	0.009
High CD4+ cells	56 (43.1)	120 (52.9)	0.075
High CD8+ cells	52 (40.3)	124 (55.1)	0.007

LDH, lactic dehydrogenase; IPI, international prognostic index; Non-GCB, Non-germinal center B cell-like; DE, double expression; DH, double-hit; TH, triple-hit. The medians were used for the cutoffs of cases with high CD68+, CD163+, CD4+ and CD8+ cells.

In our cohort, 30 VSIG4+ and 17 VSIG4- cases had COO classification results determined by Lymph2Cx. Consistent with IHC-based classification, VSIG4+ DLBCL had a significantly higher prevalence of ABC phenotype (60.0% versus 29.4%, **Supplementary Figure S5A**). ABC cases also showed a higher abundance of VSIG4+ cells than GCB and unclassified cases (**Supplementary Figure S5B**).

We examined the correlation between VSIG4 expression and the abundance of CD68+, CD163+, CD206+, CD4+ and CD8+ cells (**Table 1**). VSIG4+ cases had higher frequencies of high CD68+ cell counts (29.7% versus 8.8%), high CD163+ cell counts (23.0% versus 2.2%), CD206+ (80.3% versus 68.4%), and high CD8+ cell counts. To determine if the positive correlation between VSIG4+ and high CD8+ cell counts was influenced by the canonical TAM markers, we conducted binary logistic regression analysis (**Supplementary Table S5**). No significant correlations were observed between VSIG4 expression and cases with high CD4+ or high CD8+ cell counts. However, positive correlations were found between cases with CD206+ or high CD163+ cell counts and high CD4+ cases. Additionally, cases with CD206+, high CD68+ cell counts, and high CD163+ cell counts showed positive correlations with high CD8+ cell counts. The findings suggested that the association between VSIG4 expression and CD8+ cell numbers might be confounded by the positive correlations between VSIG4 and canonical TAM markers.

### Genetic characteristic of VSIG4+ DLBCL

**Figure 5:**
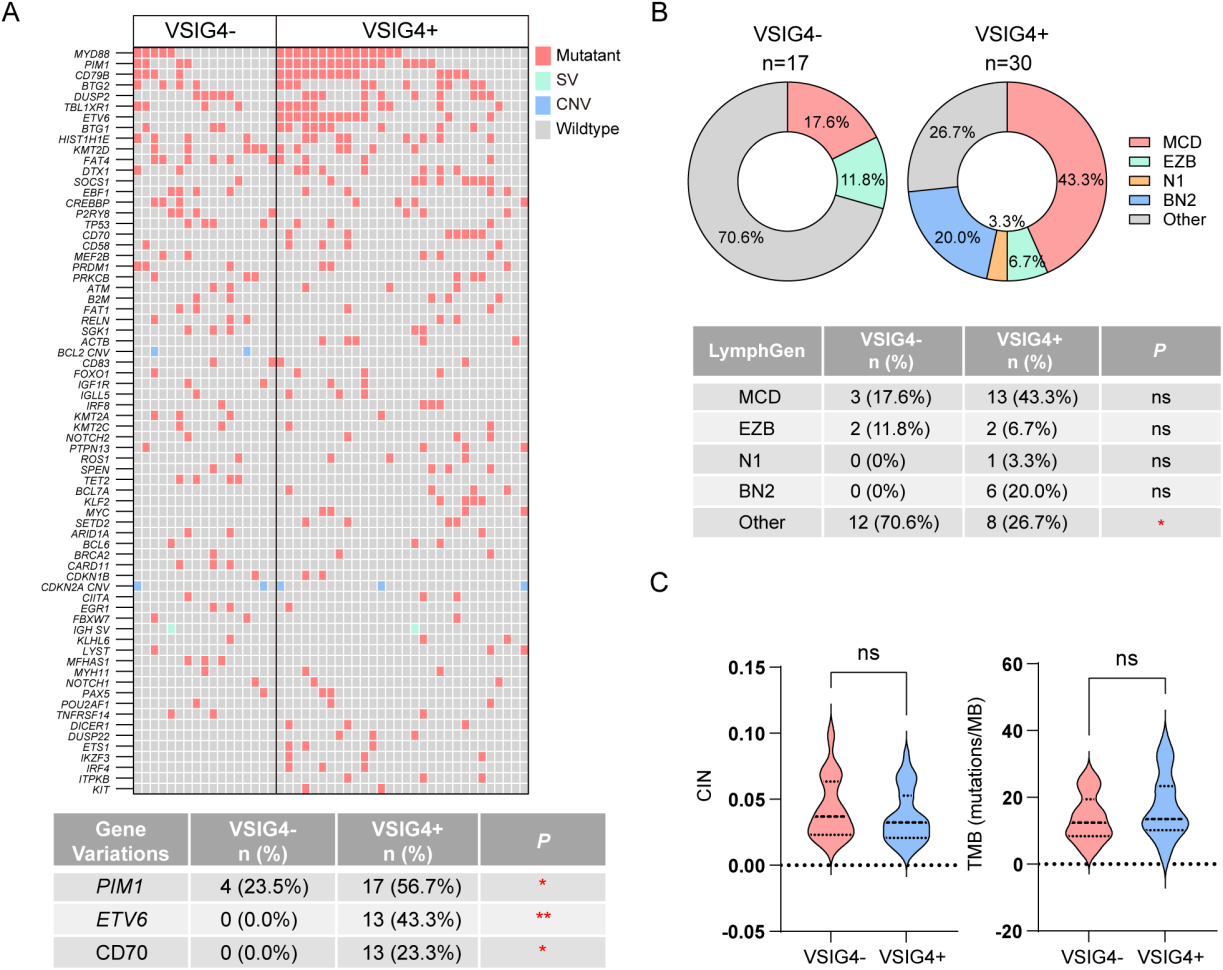
NGS-based genetic analysis and LymphGen subtyping of VSIG4+ and VSIG4- cases. **(A)** Heatmap of most frequently variated genes in VSIG4- and VSIG4+ cases and list of significantly variated genes. **(B)** Comparison of LymphGen subtyping between VSIG4+ and VSIG4- cases. **(C)** Chromosome instability scores and tumor mutation burden scores of the cases between VSIG4- and VSIG4+ group. * indicates *P*-value <0.05; ** indicates *P*-value <0.01; *** indicates *P*-value <0.001; ns indicates “not significant”.

In our study, DNA-based targeted sequencing was conducted on 30 VSIG4-expressing cases and 17 non-expressing cases to identify genetic variations, with the top 70 variations shown in **Figure 5A**. VSIG4+ group had significantly higher rates of *PIM1* (56.7% versus 23.5%), *ETV6* (43.3% versus 0.0%), and *CD70* mutations (23.3% versus 0.0%).Common mutations in VSIG4+ DLBCL also included *MYD88*, *CD79B*, *BTG2*, *DUSP2*, *TBL1XR1*, and *BTG1*. Additionally, we performed molecular subtyping using LymphGen tool, revealing a higher, though not statistically significant, proportion of MCD subtype in VSIG4+ DLBCL (43.3% vs. 17.6%, **Figure 5B**). VSIG4+ DLBCL also showed a lower rate of unclassified cases (26.7% versus 70.6%). No significant differences were observed in chromosomal instability or tumor mutational burden between VSIG4- and VSIG4+ DLBCL (**Figure 5C**).

### VSIG4+ TAMs potentially associate with T cell exhaustion

To explore the potential impact of VSIG4+ on T-cell function, we analyzed publicly available single-cell transcriptomic data from 7 DLBCL cases. Through dimensionality reduction and cell annotation, we obtained the gene expression information of tumor microenvironment cells, including TAMs, CD4+ T cells, and CD8+ T cells (**Supplementary Figure S6A**). We examined the distribution of CD68+, CD163+, and VSIG4+ subpopulations within the macrophages and found that the VSIG4+ and CD163+ subpopulations exhibited similar distribution patterns (**Supplementary Figure S6B**). Specifically, 77.05% of VSIG4+ TAMs co-expressed CD68 and CD163, while only 23.31% were CD68+/CD163− (**Supplementary Figure S6C**), which is consistent with our Multiplex IHC results. Based on the average VSIG4 expression levels, we divided the samples into VSIG4-high and VSIG4-low groups (**Supplementary Figure S6D**). Analysis of TME cell abundance between the two groups revealed no significant differences in the abundance of CD4+ TILs and CD8+ TILs (**Supplementary Figure S6E**). Furthermore, we analyzed the differential gene expression of CD4+ T cells and CD8+ T cells between the two groups (**Supplementary Figure S6F**) and utilized the AUCell tool to assess the activity of the NF-κB and JAK-STAT pathways. We found no significant differences in pathway activity between the two groups in either CD4+ T cells or CD8+ T cells (**Supplementary Figures S6G, H**). These findings suggest that VSIG4 may not act through the canonical signaling pathways in T cells.

Interestingly, we observed that the expression of the exhaustion marker TIM-3 was significantly higher in CD8+ T cells of the VSIG4-high group compared to the VSIG4-low group (**Supplementary Figure S6G**). We further employed the TCellSI tool to analyze the differences in T cell exhaustion scores between the two groups. We found that, compared to the VSIG4-low group, the VSIG4-high group had lower progenitor exhaustion scores but higher terminal exhaustion scores in CD4+ T cells, while both progenitor and terminal exhaustion scores were higher in CD8+ T cells (**Supplementary Figures S7A, B**). DEG analysis revealed that multiple immune checkpoints (LAG3, TIGIT, HAVCR2, CTLA4) and T cell exhaustion regulator (BATF, PRDM1) were upregulated in both CD4+ T cells and CD8+ T cells of the VSIG4-high group (**Supplementary Figures S7B, C**). These findings collectively suggest a potential association between VSIG4+ TAMs and T-cell exhaustion.

### VSIG4 is an independent prognostic marker for unfavorable overall survival in DLBCL

**Figure 6:**
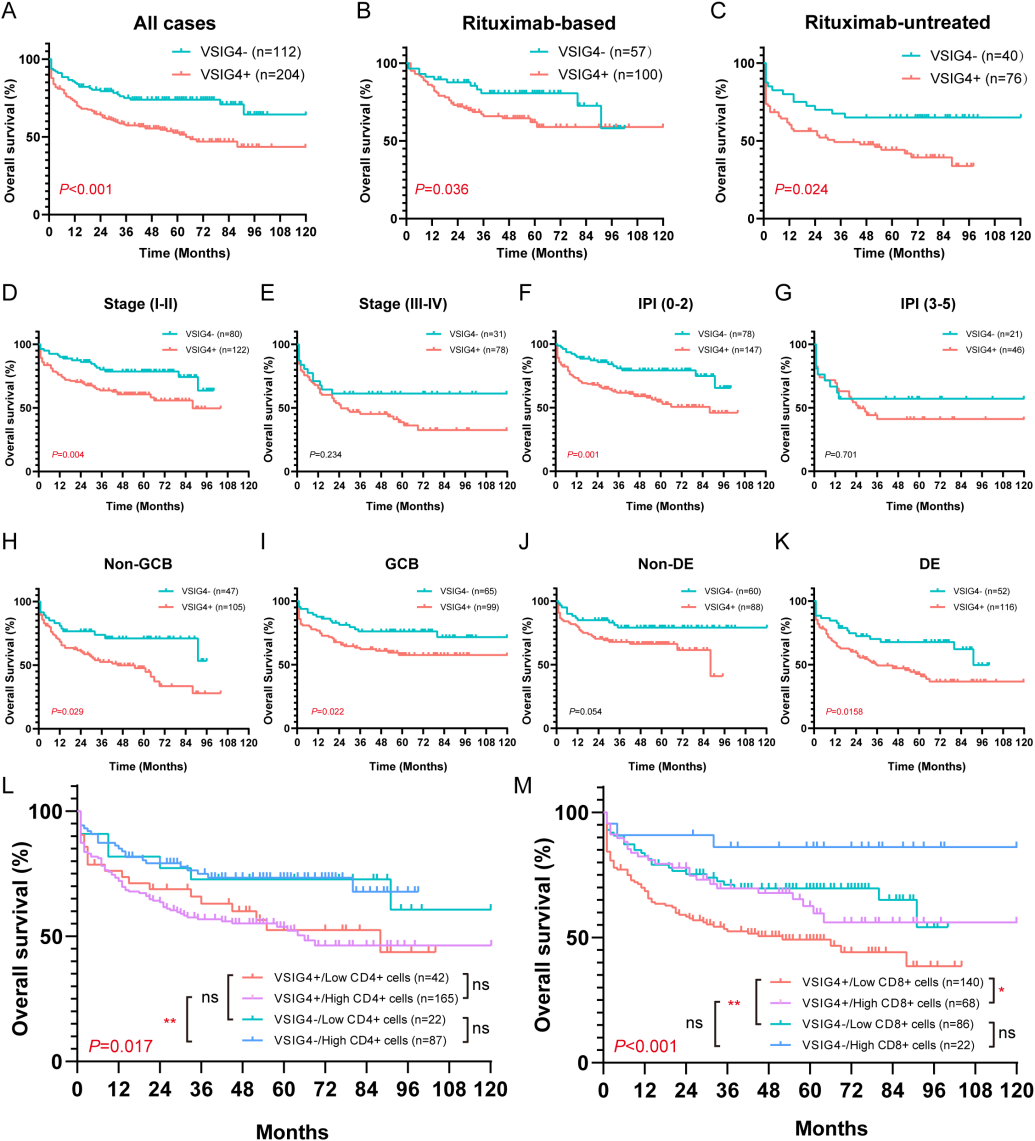
Overall survival of VSIG4- and VSIG4+ cases in different clinicopathological status. **(A)** OS of VSIG4- cases versus VSIG4+ cases. **(B, C)** OS of VSIG4- cases versus VSIG4+ cases in rituximab-based or rituximab-free group. **(D, E)** OS of VSIG4- cases versus VSIG4+ cases in early stage (I-II) or late stage (III-IV) group. **(F, G)** OS of VSIG4- cases versus VSIG4+ cases in low (0-2) or high (3-5) IPI group. **(H, I)** OS of VSIG4- cases versus VSIG4+ cases in non-GCB or GCB group. **(J, K)** OS of VSIG4- cases versus VSIG4+ cases in non-DE or DE group. **(L)** OS of cases grouped by VSIG4 expression and CD4+ cell abundance. **(M)** OS of cases grouped by VSIG4 expression and CD8+ cell abundance.

Our cohort’s follow-up period ranged from 1–120 months, with a median of 39.5 months. VSIG4+ cases exhibited significantly poor OS compared to VSIG4- cases (median survival: 64 months versus 161 months, **Figure 6A**). No statistical differences in OS were found between cases with high and low CD68+ cells, cases with high and low CD163+ cells, or cases with and without CD206+ cells, although a trend towards worse survival was noted in cases with high CD163+ cell counts (**Supplementary Figures S8A-C**).

Notably, the presence of VSIG4+ cells was found to be a predictor of poor prognosis, regardless of rituximab treatment. In both rituximab-treated and untreated groups, VSIG4-expressing cases exhibited significantly worse OS. (**Figures 6B, C**). This trend was consistent across groups with distinct clinical stages, IPI scores, COO subtypes, and DE phenotypes (**Figures 6D-K**). Univariate Cox analysis identified age (>60 years), advanced clinical stage (III-IV), high IPI score (3-5), hypoalbuminemia, presence of extranodal sites (≥2), elevated LDH, non-GCB phenotype, MYC+/BCL-2+ double-expressing phenotype, and the expression of VSIG4 as adverse prognosis factors, while rituximab as a protective factor. Multivariate Cox analysis confirmed hypoalbuminemia (HR [95% CI: 2.051-6.408]) and the expression of VSIG4 (HR [95% CI: 1.201-5.067]) as independent risk factors and rituximab use as a protective factor of OS (HR [95% CI: 0.223-0.718]) (**Table 2**).

**Table 2:** Univariate and multivariate analysis of DLBCL cohort.

Variable	Univariate		Multivariate	
	HR (95% CI)	*P*	HR (95% CI)	*P*
Age > 60 y	1.178-2.433	0.004		
Stage (III-IV)	1.344-2.711	<0.001		
IPI (3-5)	1.148-2.541	0.008		
Hypoalbuminemia	2.208-4.597	<0.001	2.051-6.480	<0.001
Extranodal sites (≥2)	1.154-2.822	0.010		
Elevated LDH	1.517-3.186	<0.001		
Non-GCB	1.001-1.008	0.021		
Use of Rituximab	0.368-0.792	0.002	0.223-0.718	0.002
MYC+/BCL-2+ DE	1.121-2.750	0.014		
VSIG4+	1.347-3.035	<0.001	1.201-5.067	0.014

IPI, international prognostic index; LDH, lactic dehydrogenase Non-GCB, Non-germinal center B cell-like; DE, double expression.

Furthermore, we noted a significantly poorer outcome in cases with lower CD8+ cell levels, whereas no significant difference was observed between cases with varying CD4+ cell levels (**Supplementary Figures S8D, E**). In cases with higher CD4+ cell counts, VSIG4 expression was associated with poor OS, though this wasn’t statistically significant in cases with low CD4+ cell counts (**Figure 6L**). Moreover, VSIG4 presence correlated with worse OS in cases with low CD8+ cell counts, while this wasn’t statistically significant in cases with high CD8+ cell counts. Notably, high CD8+ cell level seemed protective, particularly in VSIG4+ cases (**Figure 6M**). These findings implied that VSIG4’s negative effect on OS might transcend the regulation of CD4/CD8 T cell tumor immunology.

## Discussion

TAMs play a pivotal role in the tumor immune microenvironment (9). During early tumor development, chemokines enrich antitumor M1 TAMs, which support antigen presentation, effector cell activation, and tumor immunity (26). However, tumor progression induces a shift towards tumor-promoting M2 TAMs due to changes in cytokines, leading to M2 predominance (27). M2 TAMs, characterized by a CD68+/CD163+ phenotype, release cytokines that deplete tumor-infiltrating lymphocytes (TILs), inactive killer T cells and facilitate tumor immune evasion (28). In DLBCL, M2 TAM abundance correlates with treatment resistance and poor prognosis (11, 12). However, in DLBCL, a systematic study of their immune phenotype and prognostic markers is lacking.

In our study, we employed the CIBERSORT algorithm to determine the relative frequencies of tumor-infiltrating immune cells in DLBCL samples from public RNA sequencing data and stratified DLBCL cases based on TAM subtype abundance. Our analysis identified a cluster with a high M2 TAM abundance, which was associated with the worst prognosis. To probe this cluster further, we performed differential gene expression analysis against other clusters and, using LASSO, COX regression, and K-M survival analysis, found that VSIG4 expression significantly correlated with poor outcomes. These findings were subsequently validated in two separate online mRNA datasets and a local cohort of 375 cases via IHC analysis.

VSIG4, a marker highly specific to macrophages (15), is expressed in various cancers including lung (29), ovarian (30), pancreatic (31), thyroid (32), and high-grade glioma (19), where it is linked to tumor progression, poor prognosis, and immunosuppression. In this study, we constructed tissue microarrays by sampling 2 mm-diameter cores from morphologically representative intratumoral regions of FFPE surgical biopsy specimens of DLBCL for IHC analysis. Tissue cores were carefully obtained from areas exhibiting diffuse and uniform tumor cell distribution, minimizing potential sampling bias. To further avoid potential heterogeneity of VSIG4 expression, all VSIG4-positive cells within each tissue core were exhaustively counted and analyzed. The sampling and exhaustive scoring approach collectively mitigated concerns about regional bias. In our cohort, we found a positive correlation between VSIG4+ cells and CD68+ macrophage infiltration, as well as CD163+ or CD206+ cells which are indicative of M2 macrophages. Flow cytometry and multiplex IHC further showed VSIG4 co-expressed with CD68+/CD163+ M2 macrophages, though a minority was also found in CD68+/CD163- cells. It is noteworthy that the presence of CD68+/CD163- macrophages was scarcely observable within the TME of DLBCL in this study, hence we cannot conclusively exclude the existence of alternative expression patterns of VSIG4 in contexts of other tumors. Notably, the VSIG4 expression in the DLBCL TME was heterogeneous; in one VSIG4+ DLBCL case, most CD68+/CD163+ cells expressed VSIG4, while in another, only a focal subset did. This suggests VSIG4+ TAMs are a distinct subset within CD68+/CD163+ cells, but the clinical or pathological significance of different expressing patterns required further exploration using multiplex IHC in larger cohorts.

In DLBCL, macrophage infiltration is highly heterogeneous (33). Previous studies using IHC have indicated a potential link between TAMs and unfavorable prognosis in DLBCL (34, 35). Nevertheless, conflicting findings have been reported (36). These discrepancies in conclusions might be ascribed to the choice of markers employed for the classification of M1/M2 phenotype. A recent study demonstrated a significant correlation between the transcriptional characteristics of macrophages in TME of DLBCL and disease prognosis (37). This finding underscored the necessity of directing attention towards the identification of practical and informative classification markers for TAMs in DLBCL. In our results, no significant impact on prognosis was observed regarding the expression of classical TAM markers, including CD68, as well as the M2 markers CD163 and CD206. However, the expression of VSIG4, serving as a potentially distinct marker for M2 TAMs, emerged as a strong independent predictor of poor prognosis. This observation suggests that the expression of VSIG4 may serve as a critical phenotypic characteristic of TAMs strongly associated with adverse outcomes in DLBCL.

In this study, we identified distinct clinicopathological features linked to VSIG4+ DLBCL. We found a higher incidence of VSIG4+ DLBCL in older patients (>60 years), implying a link between VSIG4+ TAM abundance and patients’ immune status. Additionally, VSIG4+ DLBCL cases showed a higher prevalence of non-GCB/ABC COO subtypes, MYC+/BCL-2+ double-expression phenotypes, mutations in *PIM1*, *ETV6*, and *CD70*, and a trend towards a higher MCD subtype frequency (43.3% vs 17.6%). These findings suggest that VSIG4+ TAM infiltration may result from multiple factors, possibly related to genetic alterations and pathological phenotypes.

As reported, there was a negative correlation between endogenous VSIG4 and inflammatory state by regulating M1/M2-polarization (38, 39). Moreover, VSIG4 exhibits negative regulatory effects on T cell activity (40), demonstrated by its ability to inhibit CD8+T cell proliferation, as well as the expression of IFN-γ and T-bet (41). Within the TME, VSIG4+ TAMs demonstrate the ability to suppress tumor immunity by negatively regulating T cell activity and are considered as a promising target for tumor immunotherapy (42). T cells are important participants in tumor immunity. In DLBCL, patients with a higher number of TILs, known as “hot tumors”, had better prognoses compared to those with fewer TILs, known as “cold tumors” (43, 44). In this study, we also observed that patients with a high number of CD8+ TILs exhibited better prognosis. Interestingly, both the CIBERSORT results obtained from online databases and the IHC results from our local cohort revealed a positive association between VSIG4 and CD4+ and CD8+ TILs. However, we observed that patients with abundant CD8+ TILs had improved prognosis and noted a positive association between VSIG4 and CD4+/CD8+ TILs. Considering the potential collinearity, we subsequently performed binary logistic regression analysis on our cohort, which suggested that the association between VSIG4 and CD4+ as well as CD8+ TILs appeared to be driven by the positive correlation between classic TAM markers (CD68, CD163, CD206) and TILs. This may explain the contradiction to that the expression of VSIG4 in other solid tumors seems to have a negative correlation with TILs (20, 31, 45). Indeed, our single-cell sequencing analysis preliminarily confirmed that there is no significant correlation between VSIG4 expression and the abundance of CD4+/CD8+ T cells or NF-κB/JAK-STAT activities, but rather an association with T cell exhaustion in DLBCL. Furthermore, survival analysis suggested that the better prognosis linked to high CD8+ TILs seemed to be ameliorated by the expression of VSIG4, implying that stratifying TAMs using VSIG4 as a marker could precisely identify the subpopulation that potentially attenuates T cell function. Interestingly, the detrimental prognosis caused by VSIG4 expression may also be mitigated by the high levels of CD8+ TILs, indicating that VSIG4 could worsen DLBCL prognosis through mechanisms independent of CD8+ TIL regulation. Given the heterogeneity of TILs, further studies are needed to elucidate VSIG4’s role in DLBCL’s TME by classifying TILs precisely.

It should be noted that there are still some limitations in this study. Firstly, our IHC results were derived from a single-center cohort, which may limit the generalizability of our findings. Additionally, despite the carefully designed sampling and scoring strategy, IHC staining based on tissue microarrays may still have inherent heterogeneity. Future studies should employ multicenter and large-scale whole mount staining to substantiate our findings. Secondly, the present study primarily focused on the clinical and pathological significance of VSIG4+ TAMs in DLBCL. The cell-to-cell regulations between VSIG4+ TAMs and T cell function were descriptive, lacking in-depth cellular and molecular mechanistic validations. Further research is required to elucidate the interaction between VSIG4+ TAMs and TILs.

In conclusion, in DLBCL, VSIG4 exhibits predominant expression in CD68+/CD163+ TAMs, signifying a unique subset of M2 TAMs. The expression of VSIG4 correlates with various molecular genetic abnormalities and distinct clinical-pathological features, and functions as an independent prognostic indicator for poor outcomes. Additionally, VSIG4+ TAMs may contribute to the adverse prognosis through TAMs-mediated tumor immune mechanisms that are independent of CD8+ TILs regulation in DLBCL and may serve as a promising immune checkpoint target.

## Data Availability

The original contributions presented in the study are included in the article/**Supplementary Files**. Further inquiries can be directed to the corresponding author.
