# Supplementary material for: VSIG4 as a tumor-associated macrophage marker predicting adverse prognosis in diffuse large B-cell lymphoma
Source: Front Immunol. 2025 Jun 5;16:1567035. doi: 10.3389/fimmu.2025.1567035 (PMC12176755; doi:10.3389/fimmu.2025.1567035)
Supplement: Supplementary file 1 [file Table1.doc]

| Characteristics | | n (%) | | |  |
| --- | --- | --- | --- | --- | --- |
|  |  | Total | VSIG4-High | VSIG4-Low | *P* |
| Age  (16-90, median 61) | <60 | 113 (48.29) | 46 (38.98) | 67 (57.76) | 0.004 |
|  | ≥60 | 121 (51.71) | 72 (61.02) | 49 (42.24) |  |
| Sex | Male | 136 (58.87) | 63 (53.39) | 73 (64.60) | 0.083 |
|  | Female | 95 (41.13) | 55 (46.61) | 40 (35.40) |  |
| Ann Arbor Stage | I-II | 109 (47.39) | 53 (45.69) | 56 (49.12) | 0.602 |
|  | III-IV | 121 (52.61) | 63 (54.31) | 58 (50.88) |  |
| IPI Score | 0-2 | 126 (65.28) | 60 (61.22) | 66 (69.47) | 0.229 |
|  | 3-4 | 67 (34.72) | 38 (38.78) | 29 (30.53) |  |

Table S1. The differences in clinical pathological characteristics between VSIG4-high and VSIG4-low cases in NCICCR cohort.

IPI, international prognostic index;
