# Supplementary material for: VSIG4 as a tumor-associated macrophage marker predicting adverse prognosis in diffuse large B-cell lymphoma
Source: Front Immunol. 2025 Jun 5;16:1567035. doi: 10.3389/fimmu.2025.1567035 (PMC12176755; doi:10.3389/fimmu.2025.1567035)
Supplement: Supplementary file 2 [file Table2.docx]

| Characteristics | | n (%) | | |  |
| --- | --- | --- | --- | --- | --- |
|  |  | Total | VSIG4-High | VSIG4-Low | *P* |
| Age  (18-86, median, 63) | <60 | 186 (39.32) | 91 (37.92) | 95 (40.77) | 0.525 |
|  | ≥60 | 287 (60.68) | 149 (62.08) | 138 (59.23) |  |
| Sex | Male | 273 (62.47) | 143 (59.58) | 130 (55.79) | 0.404 |
|  | Female | 200 (37.53) | 97 (40.42) | 103 (44.21) |  |
| Ann Arbor Stage | I-II | 223 (47.15) | 106 (44.17) | 117 (50.21) | 0.188 |
|  | III-IV | 250 (52.85) | 134 (55.83) | 116 (49.79) |  |
| IPI Score | 0-2 | 277 (64.87) | 128 (60.09) | 149 (69.63) | 0.039 |
|  | 3-5 | 150 (35.23) | 85 (39.91) | 65 (30.37) |  |
| ECOG Score | 0-1 | 377 (79.70) | 186 (77.50) | 191 (81.97) | 0.226 |
|  | ≥2 | 96 (20.30) | 54 (22.50) | 42 (18.03) |  |
| LDH | Normal | 149 (34.89) | 68 (31.48) | 81 (38.39) | 0.134 |
|  | Elevated | 278 (65.11) | 148 (68.52) | 130 (61.61) |  |
| COO | GCB | 250 (52.85) | 112 (46.67) | 138 (59.23) | 0.006 |
|  | Non-GCB | 223 (47.15) | 128 (53.33) | 95 (40.77) |  |

Table S2. The differences in clinical pathological characteristics between VSIG4-high and VSIG4-low cases in GSE31312.

IPI, international prognostic index; ECOG, Eastern Cooperative Oncology Group; GCB, germinal center B cell-like,LDH, lactic dehydrogenase; COO, cell-of-origin.
