# Supplementary material for: VSIG4 as a tumor-associated macrophage marker predicting adverse prognosis in diffuse large B-cell lymphoma
Source: Front Immunol. 2025 Jun 5;16:1567035. doi: 10.3389/fimmu.2025.1567035 (PMC12176755; doi:10.3389/fimmu.2025.1567035)
Supplement: Supplementary file 3 [file Table3.docx]

Table S3. The differences in clinical pathological characteristics between VSIG4-high and VSIG4-low cases in GSE87371.

| Characteristics | | n (%) | | | *P* |
| --- | --- | --- | --- | --- | --- |
|  |  | Total | VSIG4-High | VSIG4-Low |  |
| Age  (18-86, median, 63) | <60 | 109 (49.32) | 65 (53.28) | 44 (44.44) | 0.192 |
|  | ≥60 | 112 (50.68) | 57 (46.72) | 55 (55.56) |  |
| Sex | Male | 116 (52.49) | 70 (57.38) | 46 (46.46) | 0.106 |
|  | Female | 105 (47.51) | 52 (42.62) | 53 (53.54) |  |
| Ann Arbor Stage | I-II | 71 (32.13) | 35 (28.69) | 36 (36.36) | 0.224 |
|  | III-IV | 150 (67.87) | 87 (71.31) | 63 (63.64) |  |
| IPI Score | 0-2 | 119 (53.85) | 62 (50.82) | 57 (57.58) | 0.039 |
|  | 3-5 | 102 (46.15) | 60 (49.18) | 42 (42.42) |  |
| COO | GCB | 84 (38.01) | 40 (32.79) | 44 (44.44) | 0.239 |
|  | ABC | 83 (37.56) | 52 (42.62) | 31 (31.31) |  |
|  | Unclassified | 34 (15.38) | 20 (16.39) | 14 (14.14) |  |
|  | PMBL | 20 (9.05) | 10 (8.20) | 10 (10.11) |  |

IPI, international prognostic index; COO, cell-of-origin.
