# Supplementary material for: VSIG4 as a tumor-associated macrophage marker predicting adverse prognosis in diffuse large B-cell lymphoma
Source: Front Immunol. 2025 Jun 5;16:1567035. doi: 10.3389/fimmu.2025.1567035 (PMC12176755; doi:10.3389/fimmu.2025.1567035)
Supplement: Supplementary file 4 [file Table4.docx]

Table S4. The differences in clinical pathological characteristics between VSIG4-high and VSIG4-low cases in GSE10846.

| Characteristics | | n (%) | | | *P* |
| --- | --- | --- | --- | --- | --- |
|  |  | Total | VSIG4-High | VSIG4-Low |  |
| Age  (14-92, median, 62.5) | <60 | 144(46.15) | 61(39.87) | 83(52.2) | 0.038 |
|  | ≥60 | 168(53.85) | 92(60.13) | 76(47.8) |  |
| Sex | Male | 169(56.52) | 76(51.7) | 93(61.18) | 0.124 |
|  | Female | 130(43.48) | 71(48.3) | 59(38.82) |  |
| Ann Arbor Stage | I-II | 144(46.6) | 66(43.42) | 78(49.68) | 0.323 |
|  | III-IV | 165(53.4) | 86(56.58) | 79(50.32) |  |
| ECOG Score | 0-1 | 234(75) | 105(68.63) | 129(81.13) | 0.016 |
|  | ≥2 | 78(25) | 48(31.37) | 30(18.87) |  |
| COO | GCB | 137(43.91) | 54(35.29) | 83(52.2) | 0.004 |
|  | Non-GCB | 175(56.09) | 99(64.71) | 76(47.8) |  |

ECOG, Eastern Cooperative Oncology Group‌; COO, cell-of-origin.
