# Supplementary material for: VSIG4 as a tumor-associated macrophage marker predicting adverse prognosis in diffuse large B-cell lymphoma
Source: Front Immunol. 2025 Jun 5;16:1567035. doi: 10.3389/fimmu.2025.1567035 (PMC12176755; doi:10.3389/fimmu.2025.1567035)
Supplement: Supplementary file 5 [file Table5.docx]

Table S4. Binary logistic regression analysis of the correlations between different macrophages markers and CD4+ or CD8+ cell density in DLBCL cases.

| Features | n (%) | | β | *P* | n (%) | | β | *P* |
| --- | --- | --- | --- | --- | --- | --- | --- | --- |
|  | Low  CD4+ cells | High  CD4+ cell |  |  | Low  CD8+ cell | High  CD8+ cell |  |  |
| VSIG4+ | 56 (43.1) | 120 (52.9) | 0.059 | 0.805 | 52 (40.3) | 124 (55.1) | 0.093 | 0.703 |
| CD206+ | 123 (68.0) | 146 (83.0) | 0.736 | 0.005 | 123 (69.1) | 145 (82.4) | 0.596 | 0.028 |
| High CD68+ cells | 69 (38.1) | 102 (58.0) | 0.453 | 0.140 | 56 (31.5) | 113 (64.2) | 0.890 | 0.007 |
| High CD163+ cells | 78 (43.1) | 95 (54.0) | 0.795 | 0.027 | 60 (33.7) | 112 (63.6) | 1.605 | <0.001 |

The medians were used for the cutoffs of cases with high CD68+, CD163+, CD4+ and CD8+ cells.
