# Supplementary figures and images for: VSIG4 as a tumor-associated macrophage marker predicting adverse prognosis in diffuse large B-cell lymphoma
Source: Front Immunol. 2025 Jun 5;16:1567035. doi: 10.3389/fimmu.2025.1567035 (PMC12176755; doi:10.3389/fimmu.2025.1567035)

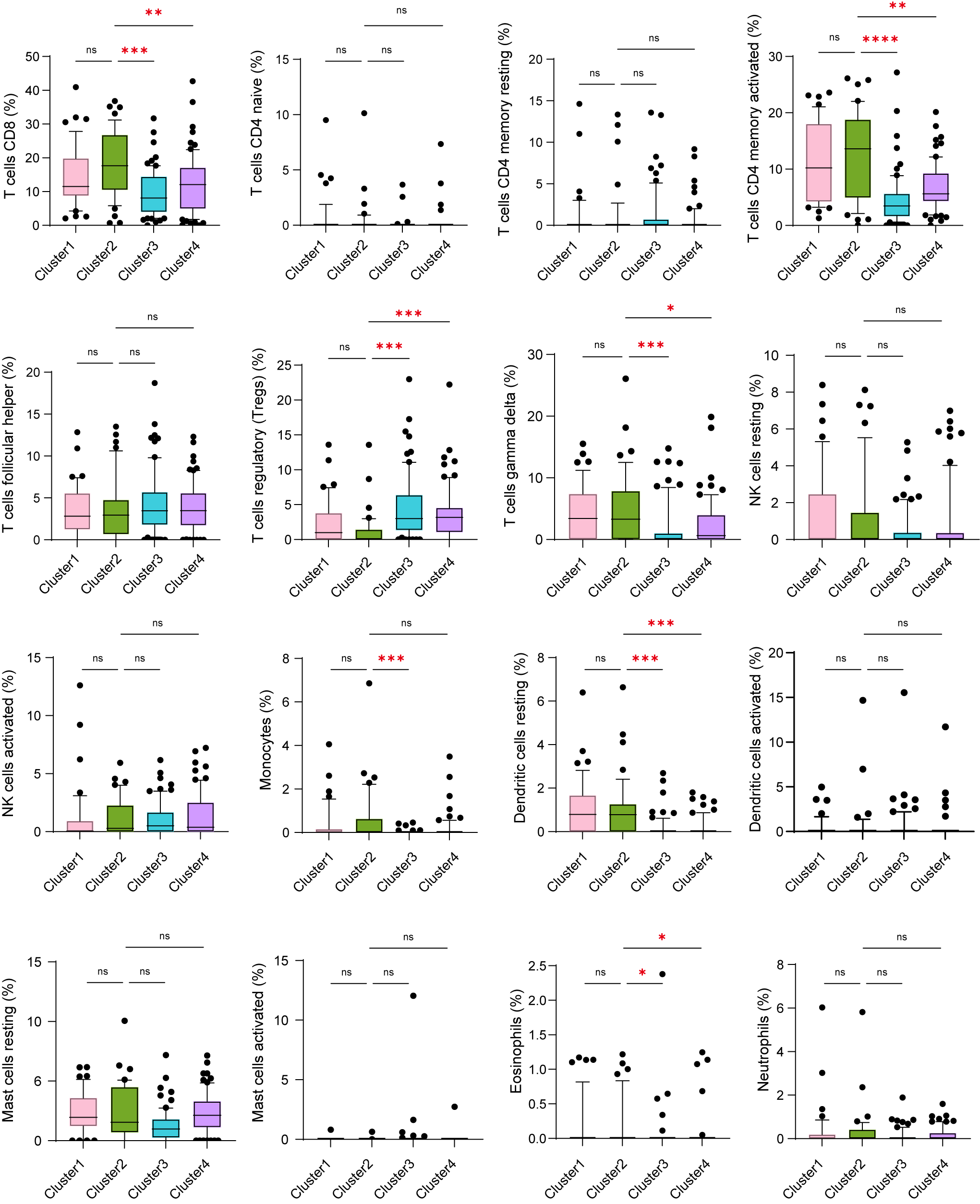

Supplement: Supplementary file 6 [file Image1.tif]

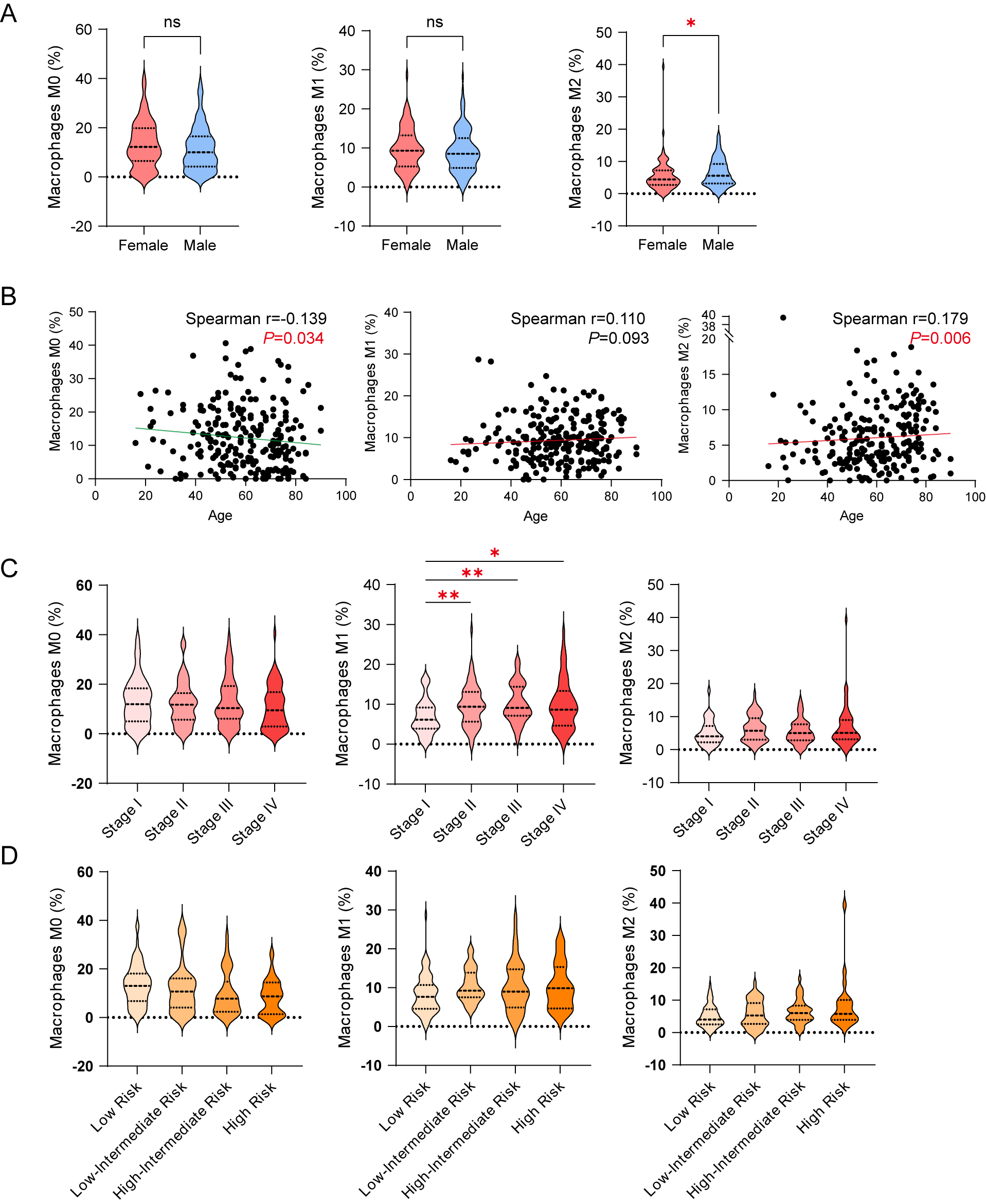

Supplement: Supplementary file 7 [file Image2.tif]

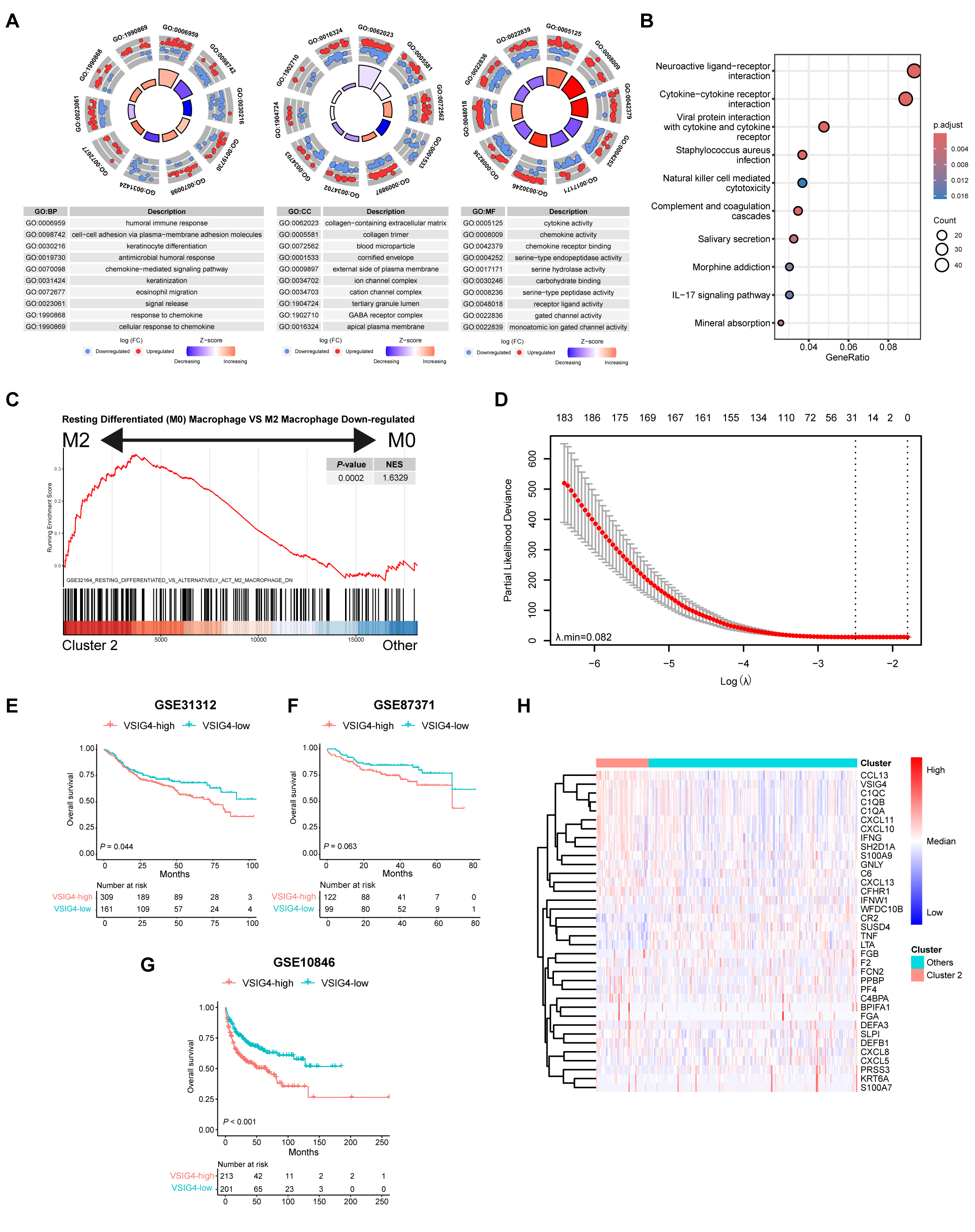

Supplement: Supplementary file 8 [file Image3.tif]

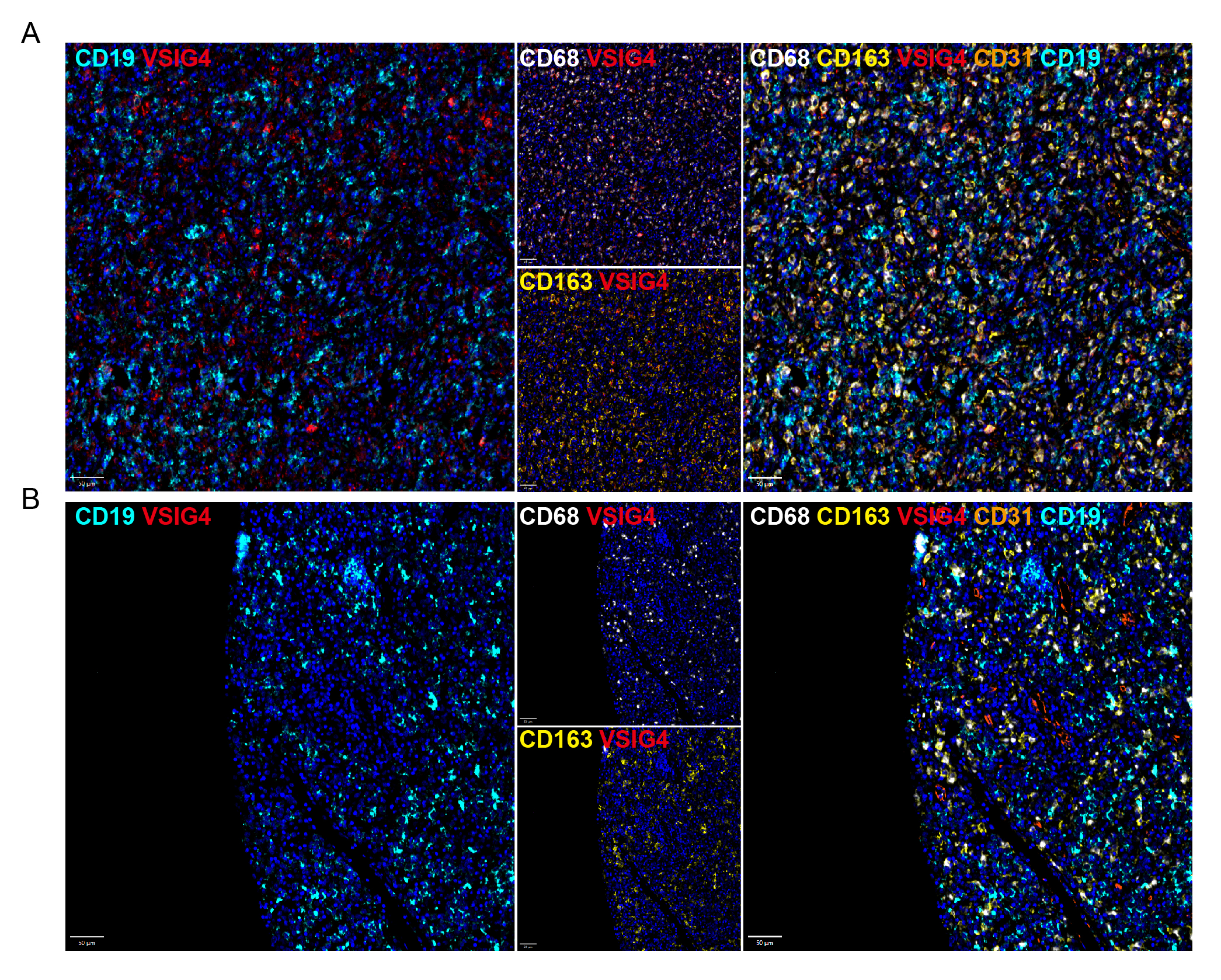

Supplement: Supplementary file 9 [file Image4.tif]

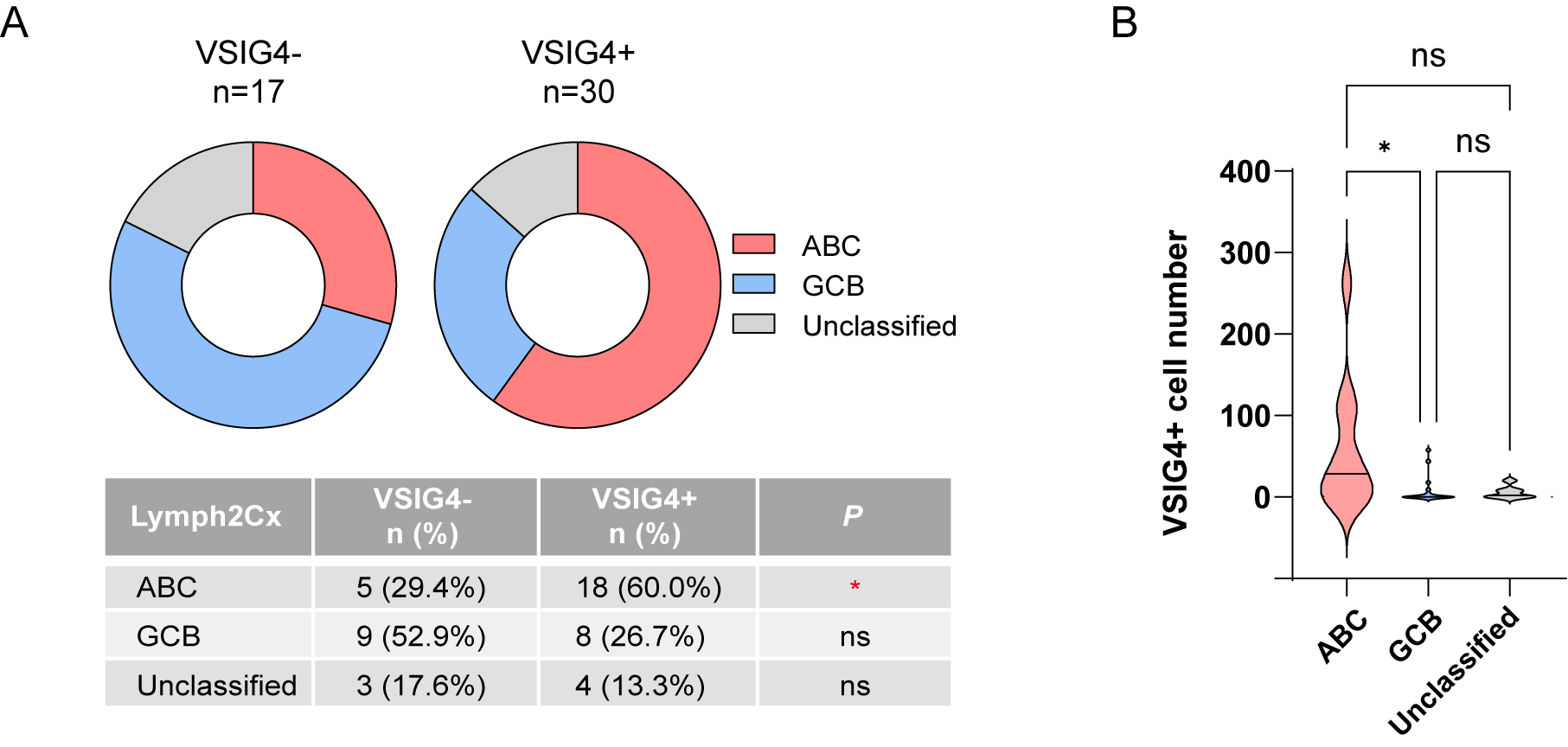

Supplement: Supplementary file 10 [file Image5.tif]

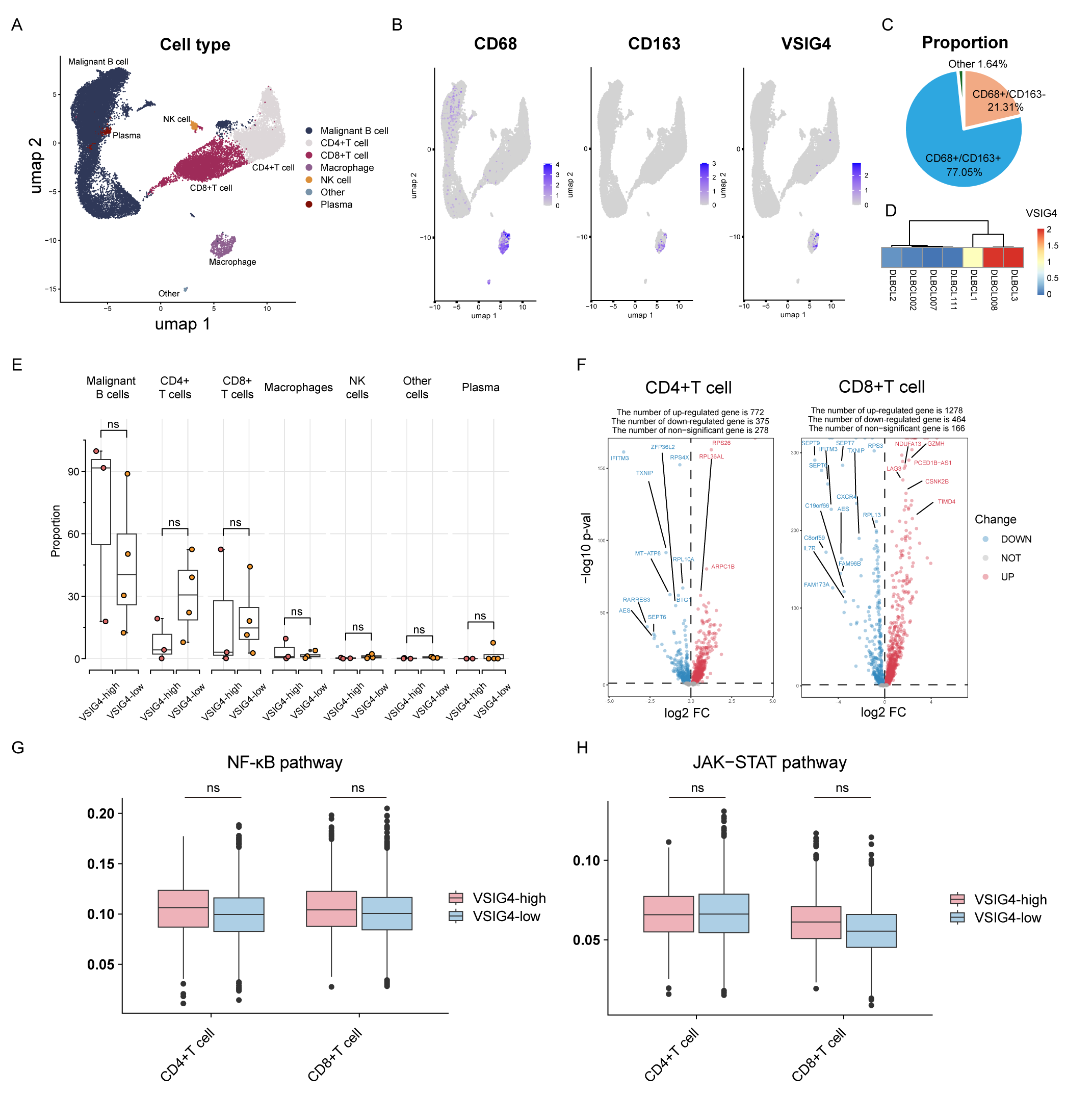

Supplement: Supplementary file 11 [file Image6.tif]

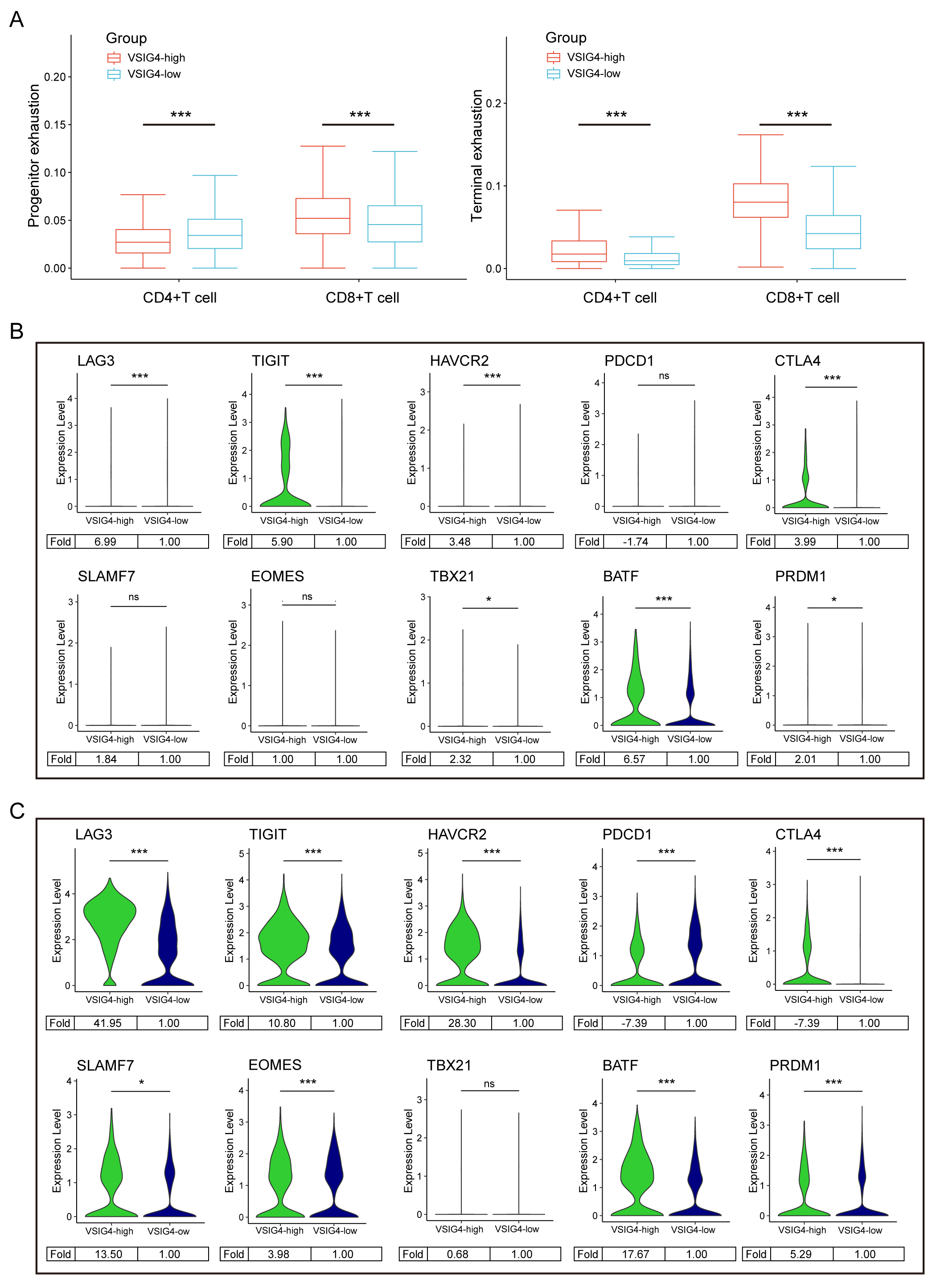

Supplement: Supplementary file 12 [file Image7.tif]

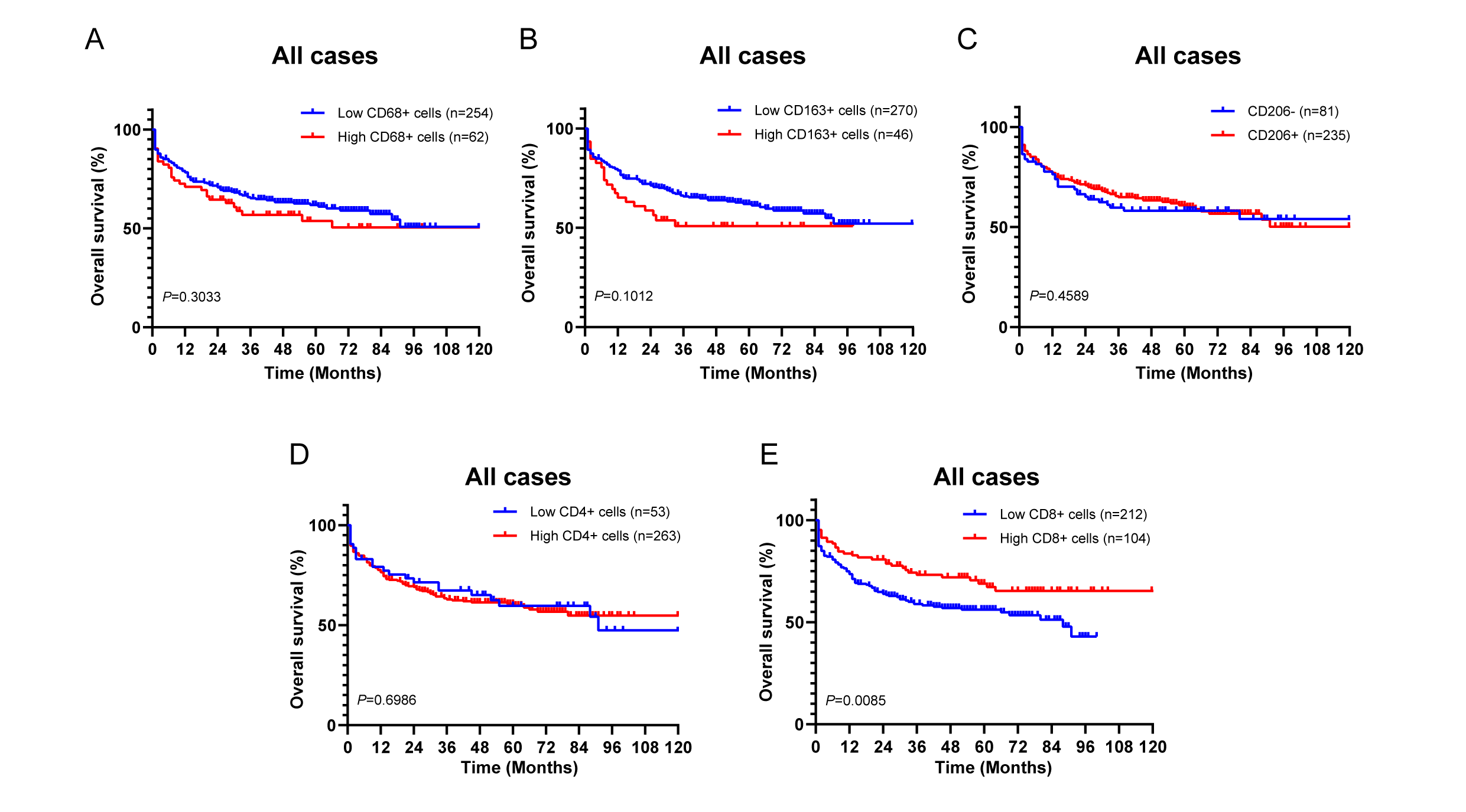

Supplement: Supplementary file 13 [file Image8.tif]
