# Supplementary material for: VSIG4 as a tumor-associated macrophage marker predicting adverse prognosis in diffuse large B-cell lymphoma
Source: Front Immunol. 2025 Jun 5;16:1567035. doi: 10.3389/fimmu.2025.1567035 (PMC12176755; doi:10.3389/fimmu.2025.1567035)
Supplement: Supplementary file 14 [file DataSheet1.docx]

**Supplementary Figure Legends**

**Figure S1.** Comparison of the abundance of infiltrating immune cells of different clusters. * indicates *P*-value <0.05; ** indicates *P*-value <0.01; *** indicates *P*-value <0.001; ns indicates “not significant”.

**Figure S2.** (A) Abundance of infiltrating macrophages (M0, M1, M2) in female or male patients. (B) Correlations between infiltrating macrophages (M0, M1, M2) with age. (C) Abundance of infiltrating macrophages (M0, M1, M2) in cases with different clinical stages. (D) Abundance of infiltrating macrophages (M0, M1, M2) with different IPI risk levels. * indicates *P*-value <0.05; ** indicates *P*-value <0.01; *** indicates *P*-value <0.001; ns indicates “not significant”.

**Figure S2.** (A) Abundance of infiltrating macrophages (M0, M1, M2) in female or male patients. (B) Correlations between infiltrating macrophages (M0, M1, M2) with age. (C) Abundance of infiltrating macrophages (M0, M1, M2) in cases with different clinical stages. (D) Abundance of infiltrating macrophages (M0, M1, M2) with different IPI risk levels. * indicates *P*-value <0.05; ** indicates *P*-value <0.01; *** indicates *P*-value <0.001; ns indicates “not significant”.

**Figure S3.** (A) The mostly enriched GO terms in cluster 2 versus other groups. (B) The mostly enriched KEGG terms in cluster 2 versus other groups. (C) GSEA analysis showing cluster 2 expressed genes which involved in M0-M2 polarization. (D) Dynamic curve of partial-likelihood deviance versus Log (lambda) in Lasso regression. (E-F) K-M plots and survival analysis of VSIG4-high group versus VSIG4-low group in validation cohort GSE31312 and GSE87371. (G) Heatmap of the expressions of the genes in GO:0006959 (humoral immune response) between cluster 2 and other clusters.

**Figure S4.** (A) Multiple IHC images of a representative case with VSIG4 expressing in almost all CD68+/CD163+ cells. (B) Multiple IHC images of a representative VSIG4- case. Original magnification ×400

**Figure S5.** (A) Comparison of Lymph2Cx-based COO between VSIG4- and VSIG4+ cases. (B) Abundance of VSIG4+ cells in cases with different COO. * indicates *P*-value <0.05; ** indicates *P*-value <0.01; *** indicates *P*-value <0.001; ns indicates “not significant”.

**Figure S6.** (A) Cell annotation of single-cell transcriptomic data of 7 DLBCL cases represented by UMAP. (B) Distribution of CD68+, CD163+ or VSIG4+ cells represented by UMAP. (C) The proportion of VSIG4+ subpopulation in CD68+/CD163+ TAMs, CD68+/CD163- TAMs or other phenotypic TAMs. (D) Case grouping based on VSIG4 expression levels. (E) Cell abundance between VSIG4-high and VSIG4-low group. (F) DEG analysis of CD4+ T cells and CD8+ T cells between VSIG4-high and VSIG4-low group represented by volcano plot. (G) Activity scoring of NF-κB pathway in CD4+ T cells and CD8+ T cells between VSIG4-high and VSIG4-low group. (H) Activity scoring of JAK-STAT pathway in CD4+ T cells and CD8+ T cells between VSIG4-high and VSIG4-low group. ns indicates “not significant”.

**Figure S7.** (A) Scoring of progenitor/terminal exhaustion in CD4+ T cells and CD8+ T cells between VSIG4-high and VSIG4-low group. (B) The expression of immune checkpoints and exhaustion regulators in CD4+ T cells between VSIG4-high and VSIG4-low group. (C) The expression of immune checkpoints and exhaustion regulators in CD8+ T cells between VSIG4-high and VSIG4-low group. * indicates *P*-value <0.05; ** indicates *P*-value <0.01; *** indicates *P*-value <0.001; ns indicates “not significant”.

**Figure S8.** (A-E) OS of cases (A) with low or high infiltrating CD68+ cells, (B) with low or high infiltrating CD163+ cells, (C) with or without infiltrating CD206+ cells, (D) with low or high CD4+ cells and (E) with low or high CD8+ cells.
