# Supplementary material for: VSIG4 as a tumor-associated macrophage marker predicting adverse prognosis in diffuse large B-cell lymphoma
Source: Front Immunol. 2025 Jun 5;16:1567035. doi: 10.3389/fimmu.2025.1567035 (PMC12176755; doi:10.3389/fimmu.2025.1567035)
Supplement: Supplementary file 15 [file DataSheet2.docx]

**Supplementary Methods**

**Bioinformatic Study**

To discover the key prognostic TAM marker, NCICCR-DLBCL (https://portal.gdc.cancer.gov/projects/NCICCR-DLBCL), an online dataset from the project of Center for Cancer Genomics (CCG) was downloaded, in which 234 cases with survival information and RNA-sequencing data were used as discovery cohort. 2 datasets, GSE31312 and GSE87371 from GEO database were used as validation cohorts.

All bioinformatic studies were processed in R software. The FPKM values and COUNTs values of protein-coding genes were normalized using “limma” package and prepared as gene expression matrices. CIBERSORT method (LM22 signature matrix was used for deconvolution) was applied to FPKM matrix to obtain the percentage of immune cells (including tumor/non-tumor B cells) in the tumor microenvironment of each case. The percentage of infiltrating macrophages of 3 subtypes (M0, M1 and M2) were then subjected to clustering analysis using “ConsensusClusterPlus” package to generate potential clusters classified by TAMs. The principal component analysis (PCA) was conducted using “prcomp” function.

The COUNTs matrix of cases in each cluster were then used for differentially expression analysis which was performed using “DESeq2” package. The differentially expressed genes (DEGs) were then applied to LASSO regression using “glmnet” package to eliminate collinearity. Next, univariate, and multivariate Cox regression analysis, followed by Kaplan-Meier (K-M) analysis, were performed by “survival” package to obtain key genes associated with prognosis. The K-M curves were plotted by “survminer” package.

Gene Set Enrichment Analysis (GSEA) including Gene oncology (GO) and Kyoto Encyclopedia of Genes and Genomes (KEGG) analysis were performed using “clusterProfiler” package and visualized by using “enrichplot” package. The Spearman correlation analysis was performed using “Hmisc” package and visualized by using “corrplot” package.

Otherwise mentioned, the bioinformatic results were visualized using “ggplot2” package, and the statistical analysis was performed and visualized using GraphPad Prism 10.

**Single-cell Transcriptome Analysis**

To investigate the potential relationship between VSIG4+ TAMS and T-cell function, public single-cell transcriptome data were collected. Data from 7 DLBCL cases were obtained from GEO (GSE182436) and heiDATA (VRJUNV), all generated by 10× Genomics technology. Quality control was performed on raw gene-cell barcode matrices using the following criteria: mitochondrial gene content (≤25%), erythrocyte gene content (≤25%), and gene counts (retained 200–10,000 genes per cell). After quality filtering, 26,647 cells were retained for downstream analysis. All following analyses were performed in R software.

For dimensionality reduction and clustering of single-cell RNA sequencing (scRNA-seq) data, scRNA-seq data were processed using the “Seurat” package (v.5.0.3) (1). Data normalization was performed with the “NormalizeData” function, followed by scaling using “ScaleData” function. The top 2,000 highly variable genes were identified via “FindVariableFeatures” function. PCA was conducted using “RunPCA” function, and batch effects were corrected using Harmony package (v1.2.0). The first 30 principal components (determined by elbow plot analysis) were selected for uniform manifold approximation and projection (UMAP) using “RunUMAP” function. Unsupervised clustering was performed at a resolution of 0.8 with “FindClusters” function.

Cell populations were annotated using marker genes from the CellMarker database and published references: B cells (CD79A, CD79B), plasma cells (JCHAIN, IGHG1, MZB1), NK cells (NKG7, GNLY), macrophages (APOE, CD68), CD8+ T cells (CD3D, CD3E, CD8A), and CD4+ T cells (CD3D, CD3E, CD4). Malignant B cells were identified using inferCNV package (v1.14.0) with CD4+ T cells as reference. Analysis parameters included HMM-based copy number variation (CNV) inference (analysis_mode = "subclusters"), and a noise-reduction threshold (cutoff = 0.1). Regions with significant CNV deviations were classified as malignant. To assess VSIG4 expression, macrophage-specific VSIG4 expression was averaged per sample using “AverageExpression” function in “Seurat” package. Samples were stratified into VSIG4-high (DLBCL1, DLBCL2, DLBCL008; mean expression ≥0.76) and VSIG4-low groups (DLBCL3, DLBCL002, DLBCL007, DLBCL111).

For T cell exhaustion scoring, Precursor and terminal exhaustion states in CD4+ and CD8+ T cells were evaluated using TCellSI package (v3.5.1) (2). Differential expressions of T cell exhaustion-related genes were assessed using Wilcoxon tests with Benjamini-Hochberg correction and visualized using “ggplot2” package. For pathway activity analysis, AUCell scoring method was used to analyze NF-κB and JAK-STAT signaling pathway. Gene set activity was quantified using “AUCell” package (v1.24.0) (3) with variable “aucMaxRank” set to the top 5% of expressed genes.

**Fluorescence *in-situ* Hybridization**

Fluorescence *in-situ* hybridization (FISH) was performed to detect MYC, BCL2, and BCL6 translocations following routine procedure. MYC, BCL2, and BCL6 break-apart probes were purchased from Abbott (USA). The slides were assessed by 2 pathologists by randomly counting 100 tumor cells and translocations were considered positive when break-apart signals were confirmed in more than 20% of counted cells.

**Multiplex Immunohistochemistry**

For multiplex IHC, multiple-staining assay was employed using a 7-color multiplex IHC kit (KR Pharmtech, China) according to the manufacturer’s instructions. Briefly, analysis was conducted on 3 mm thick formalin-fixed paraffin-embedded (FFPE) slides. The slides were dewaxed, rehydrated, and subjected to heat retrieval using EDTA or sodium citrate buffer. The slides were blocked using Antibody Diluent Block Buffer, and incubated using the primary antibody for 40-60 minutes, followed by a polymer HRP-conjugated antibody for 10 minutes. After washing, Fluorophore Working Solution was added and incubated for 10 minutes to generate the signal. All incubations were performed at room temperature. Nuclei were stained with DAPI after all the antigens above have been labeled. Once all target proteins were stained, whole slides were scanned at ×20 using the KR-HT5 system (KR Pharmtech, China). The anti-bodies used in this method included: VISG4 (Abcam, Clone: EPR22576-70, Cat.AB252933); CD31(ZSGB-BIO, Clone: EP78, Cat.ZA-0568); CD19(ZSGB-BIO, Clone: UMAB103, Cat.ZM-0038, ready-to-use); CD163(Abcam, Clone: EPR19518, Cat.AB182422) and CD68(Abcam, ab955, 1:6000).

**Flowcytometry**

Flowcytometry of lymph nodes was performed on 3 DLBCL cases following routine procedure. In brief, freshly isolated lymph nodes from DLBCL patients were injected with RPMI 1640 culture medium (Thermo Fisher, USA) with 10% fetal bovine serum and gently grinded in a cold dish to release the cells inside. Then, the cells were collected, washed, and incubated in a 4-color antibody cocktail consisting of VSIG4-Alexa Fluor 488 (Thermo Fisher, Cat.53-5757-42, USA), CD19-PE-Cy5.5 (Beckman Coulter, Cat.A66328, USA), CD68-PE (BioLegend, Cat.333807, USA) and CD163-PE-Cy7 (BioLegend, Cat.326513, USA). The flowcytometry was performed on a Cytek Spectrum Flowcytometer (NL-CLC, V16-B14, USA).

**Cell-of-origin Analysis by Lymph2Cx**

COO was determined by Lymph2Cx assay in 47 cases of the local cohort (NanoString Technologies, USA). In brief, RNA was extracted from FFPE samples and hybridized to the Lymph2Cx CodeSet on the nCounter PrepStation under the “high sensitivity” setting and analyzed with the nCounter Analyzer. The mRNA counts of 15 lymphoma-related genes were determined and normalized by the mean of 5 housekeeping genes to calculate the predictor scores for COO subtyping.

**Next Generation Sequencing and LymphGen Subtyping**

The DNA-based targeted sequencing was performed in 47 cases. The FFPE sections were reviewed for sufficient tumor abundance (>30%). After DNA extraction and fragmentation using a QIAamp DNA FFPE Tissue Kit (QIAGEN, Germany) and a Bioruptor (Diagnede, Belgium), the libraries were prepared with KAPA Hyper DNA Library Prep Kit (Roche, Switzerland) and then were subjected to hybrid selection process. The probes used for hybrid selection which targeting 475 leukemia- and lymphoma-related genes were designed by GENESEEQ Technology (China). Sequencing was performed as 150-bp reads using a HiSeq 4000 instrument (Illumina, USA). The bioinformatics analysis of sequencing results was performed as previously described (4). The molecular subtyping of DLBCL cases were performed using LymphGen on-line classifier (<http://llmpp.nih.gov/lymphgen/index/php>).

**Statistical Analysis**

All statistical evaluations for local cohort were conducted using SPSS 26.0 (IBM, USA) and GraphPad Prism 10 software (La Jolla, USA). Clinical and pathological characteristics between the 2 groups were compared by using χ^2^ test, Fisher’s exact test or Mann-Whitney *U* test. Binary logistic regression was used to analyze the correlation of each feature and T-cell abundance. The K-M analysis was used to establish survival curved of each group. Log-rank tests were used to compare the overall survival (OS) of each group. The cutoff values of CD68+ cells, CD163+ cells, CD4+ cells and CD8+ cells for survival analysis were derived from X-tile 3.6.1 software (Yale, USA). The univariate and multivariate Cox proportional hazards models were used to find independent risk factors.

1. Hao Y, Stuart T, Kowalski MH, Choudhary S, Hoffman P, Hartman A, et al. Dictionary Learning for Integrative, Multimodal and Scalable Single-Cell Analysis. *Nature biotechnology* (2024) 42(2):293-304. Epub 2023/05/26. doi: 10.1038/s41587-023-01767-y.

2. Yang JM, Zhang N, Luo T, Yang M, Shen WK, Tan ZL, et al. Tcellsi: A Novel Method for T Cell State Assessment and Its Applications in Immune Environment Prediction. *iMeta* (2024) 3(5):e231. Epub 2024/10/21. doi: 10.1002/imt2.231.

3. Van de Sande B, Flerin C, Davie K, De Waegeneer M, Hulselmans G, Aibar S, et al. A Scalable Scenic Workflow for Single-Cell Gene Regulatory Network Analysis. *Nature protocols* (2020) 15(7):2247-76. Epub 2020/06/21. doi: 10.1038/s41596-020-0336-2.

4. Ma D, Ma Y, Ma Y, Liu J, Gu Y, Liu N, et al. Molecular Subtyping of Cd5+ Diffuse Large B-Cell Lymphoma Based on DNA-Targeted Sequencing and Lymph2cx. *Front Oncol* (2022) 12:941347. Epub 2022/09/10. doi: 10.3389/fonc.2022.941347.
